# Supplementary material for: Cross-reactivity of glycan-reactive HIV-1 broadly neutralizing antibodies with parasite glycans
Source: Cell Rep. 2022 Mar 29;38(13):110611. doi: 10.1016/j.celrep.2022.110611 (PMC10073069; doi:10.1016/j.celrep.2022.110611)
Supplement: Document S1. Figures S1–S7 and Tables S1 and S2 [file mmc1.pdf]

Cell Reports, Volume *38*

Supplemental information

Cross-reactivity of glycan-reactive HIV-1 broadly neutralizing antibodies with parasite glycans

Isabella Huettner, Stefanie A. Krumm, Sonia Serna, Katarzyna Brzezicka, Serena Monaco, Samuel Walpole, Angela van Diepen, Fiona Allan, Thomas Hicks, Simon Kimuda, Aidan M. Emery, The IAVI Protocol C Investigators & The IAVI African HIV Research Network, Elise Landais, Cornelis H. Hokke, Jesus Angulo, Niels Reichardt, and Katie J. Doores

Supplementary information

Cross-reactivity of glycan-reactive HIV-1 broadly neutralizing antibodies with parasite glycans.

Isabella Huettner,[1] Stefanie A. Krumm,[1] Sonia Serna,[2] Katarzyna Brzezicka,[2] Serena Monaco,[3] Samuel Walpole,[3] Angela van Diepen,[4] Fiona Allan,[5] Thomas Hicks,[3] Simon Kimuda,[1] Aiden M. Emery,[5] The IAVI Protocol C Investigators & The IAVI African HIV Research Network**, Elise Landais,[6,7] Cornelis H. Hokke,[4] Jesus Angulo,[8] Niels Reichardt,[2,9] Katie J. Doores[1,10]*

[1] Department of Infectious Diseases, School of Immunology & Microbial Sciences, King's College London, London, UK.

[2] Glycotechnology Laboratory, Center for Cooperative Research in Biomaterials (CIC biomaGUNE), Basque Research and Technology Alliance (BRTA), Paseo Miramón 182, 20014 San Sebastian, Spain.

[3] School of Pharmacy, University of East Anglia, Norwich Research Park, Norwich, Norfolk NR4 7TJ, UK.

[4] Department of Parasitology, Leiden University Medical Center, Leiden, the Netherlands.

[5] Department of Life Sciences, Natural History Museum, Cromwell Road, London, United Kingdom.

[6]International AIDS Vaccine Initiative Neutralizing Antibody Center, La Jolla, CA 92037, USA.

[7]International AIDS Vaccine Initiative, New York, NY 10004, USA.

[8]Departamento de Química Orgánica, Universidad de Sevilla, C/ Prof. García González, 1, 41012 Sevilla, Spain; Instituto de Investigaciones Químicas (CSIC-US), Avda. Américo Vespucio, 49, 41092 Sevilla, Spain.

[9]CIBER-BBN, Paseo Miramón 182, 20009 San Sebastian, Spain

[10]Lead contact

* Corresponding author: katie.doores@kcl.ac.uk

**List of investigators and details of affiliations are listed in the acknowledgements

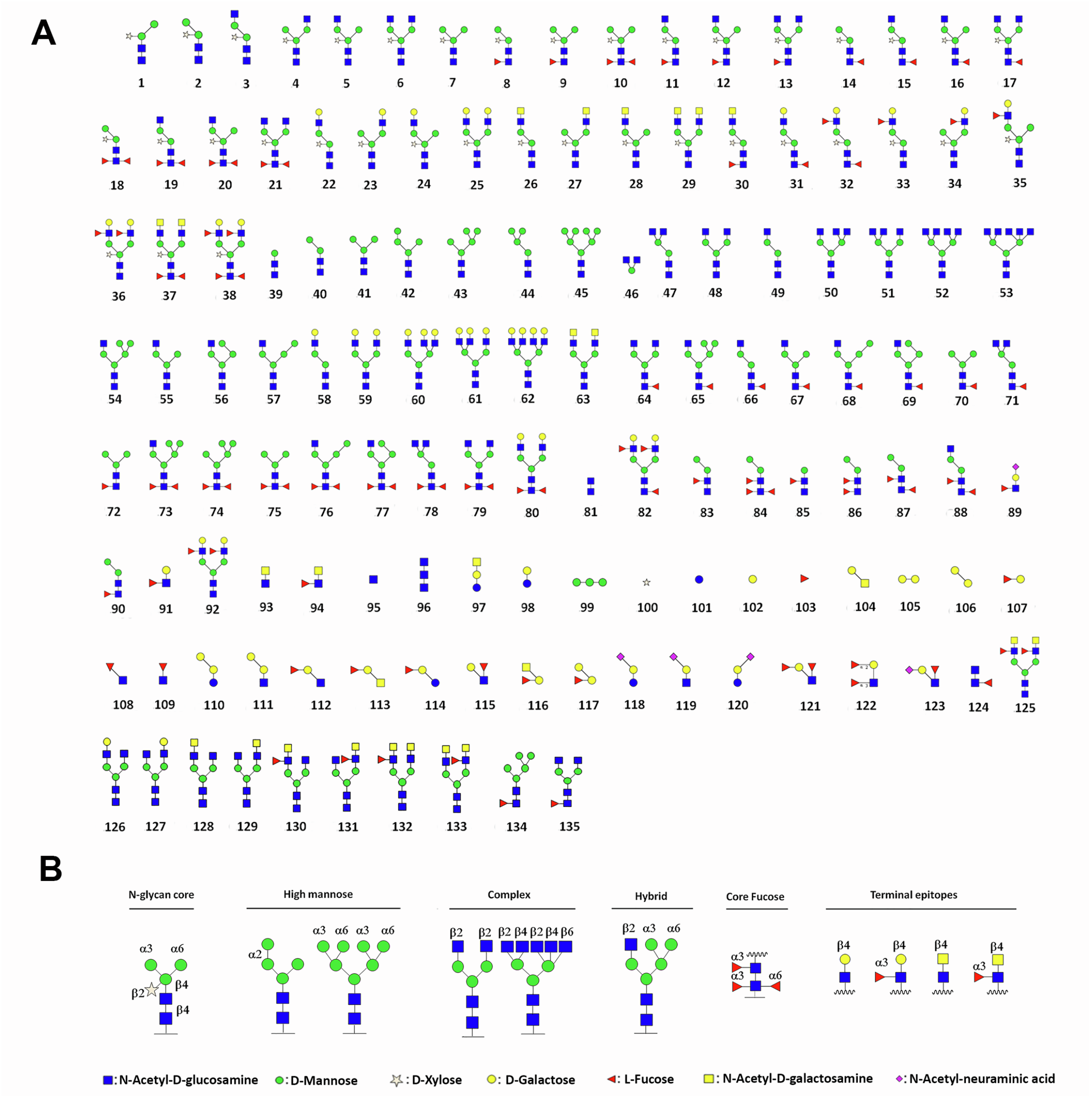

Figure S1: Glycan structures present on the synthetic glycan microarray. A) List of glycan structures present on the synthetic glycan array used in **Figure 1A** and **Figure 2E**. The array used in **Figure 1A** had glycans 1-125 and the array used in **Figure 2E** had been developed further and includes glycans 1-135. **B)** Key for glycan structures shown in panel A. Man: green circle, GlcNAc: blue square, GalNAc: yellow square, Gal: yellow circle, Sialic acid: purple diamond, Fucose: red triangle, and xylose: star. This figure relates to **Figure 1** and **Figure 2E**.

A

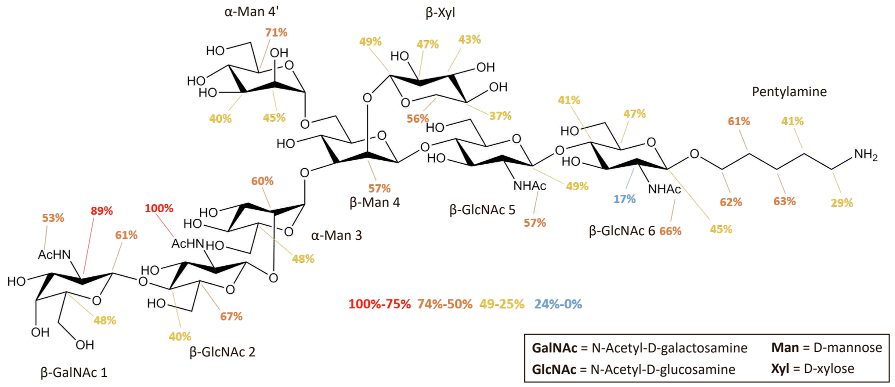
α-Man 4'
β-Xyl
Pentylamine
β-Man 4
α-Man 3
β-GlcNAc 5
β-GlcNAc 6
β-GlcNAc 2
β-GalNAc 1
100%-75% 74%-50% 49-25% 24%-0%
GalNAc = N-Acetyl-D-galactosamine
GlcNAc = N-Acetyl-D-glucosamine
Man = D-mannose
Xyl = D-xylose

B

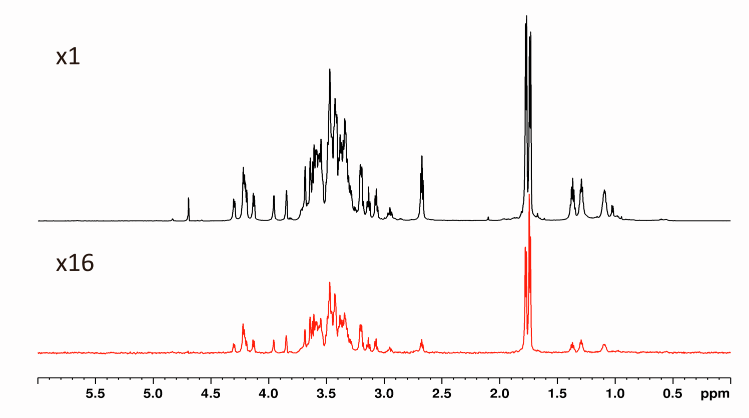
x1
x16
5.5 5.0 4.5 4.0 3.5 3.0 2.5 2.0 1.5 1.0 0.5 ppm

C

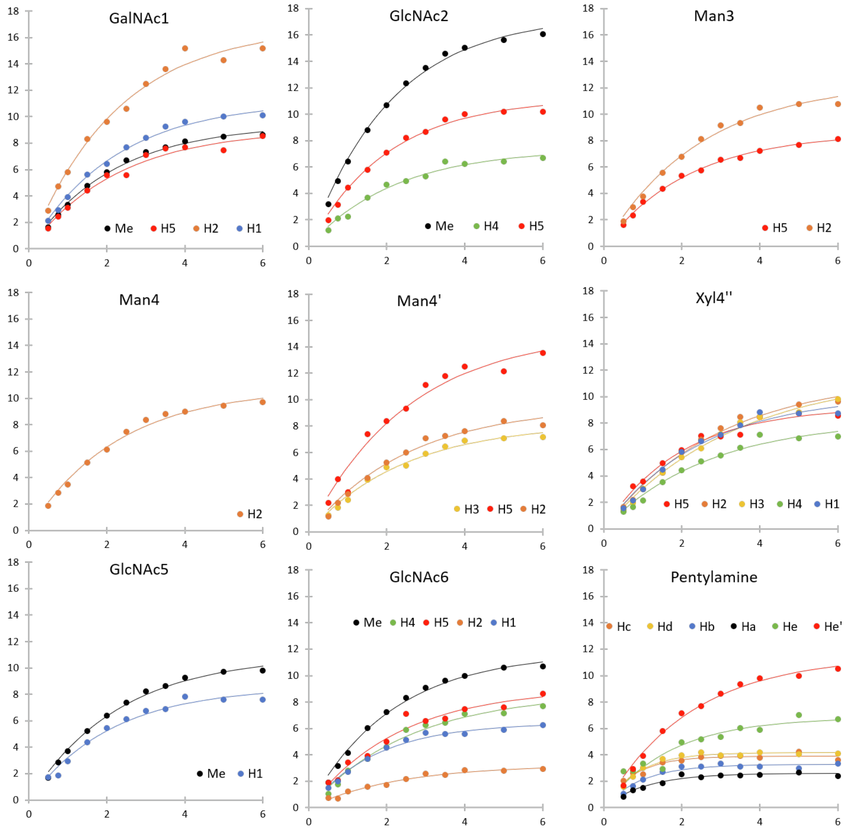
GalNAc1
GlcNAc2
Man3
Man4
Man4'
Xyl4''
GlcNAc5
GlcNAc6
Pentylamine
Me H5 H2 H1
Me H4 H5
H5 H2
H2
H3 H5 H2
H5 H2 H3 H4 H1
Me H1
Me H4 H5 H2 H1
Hc Hd Hb Ha He He'

Figure S2: A) Binding epitope mapping of the parasite glycan 28 as bound to PGT121. Binding epitope mapping of glycan **28** as bound to PGT121 was obtained by mathematically fitting the experimental STD NMR build up curves (see below) to a mono-exponential equation and following an initial slopes analysis. The binding epitope was obtained by normalising all by the largest value, to which an arbitrary value of 100 % was assigned. Hence, higher relative STD % values correspond to closer glycan contacts with PGT121 in the bound state. **B) STD NMR spectra of the glycan 28 / PGT121 complex in solution.** Reference (black) and difference (red) spectra of glycan **28** in the presence of PGT121 at 2 s saturation time. **C) STD NMR build-up curves of the complex of PGT121 with glycan 28**, where the saturation time is represented on the x-axis and the STD% is represented on the y-axis. Build-up curves are obtained for each non-exchangeable hydrogen atom, and they are here grouped by sugar residues. This figure relates to **Figure 2**.

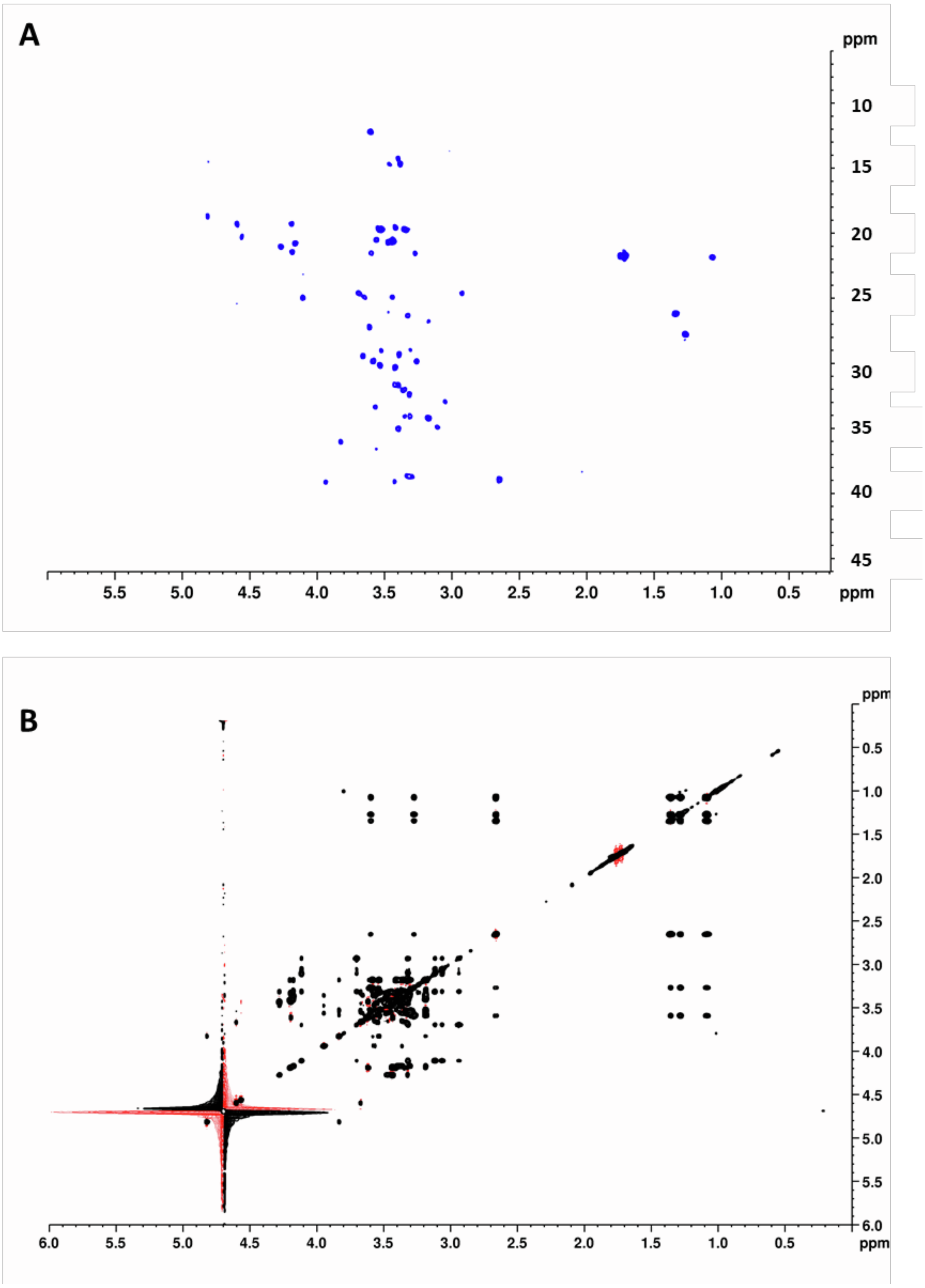

Figure S3: A) HSQC (*hsqcetgpsp*) and B) TOCSY (*mlvevphpp*) of glycan 28 in D_2O, based on which the assignment was based. See Table 2 for full list of assignment. This figure relates to **Figure 2**.

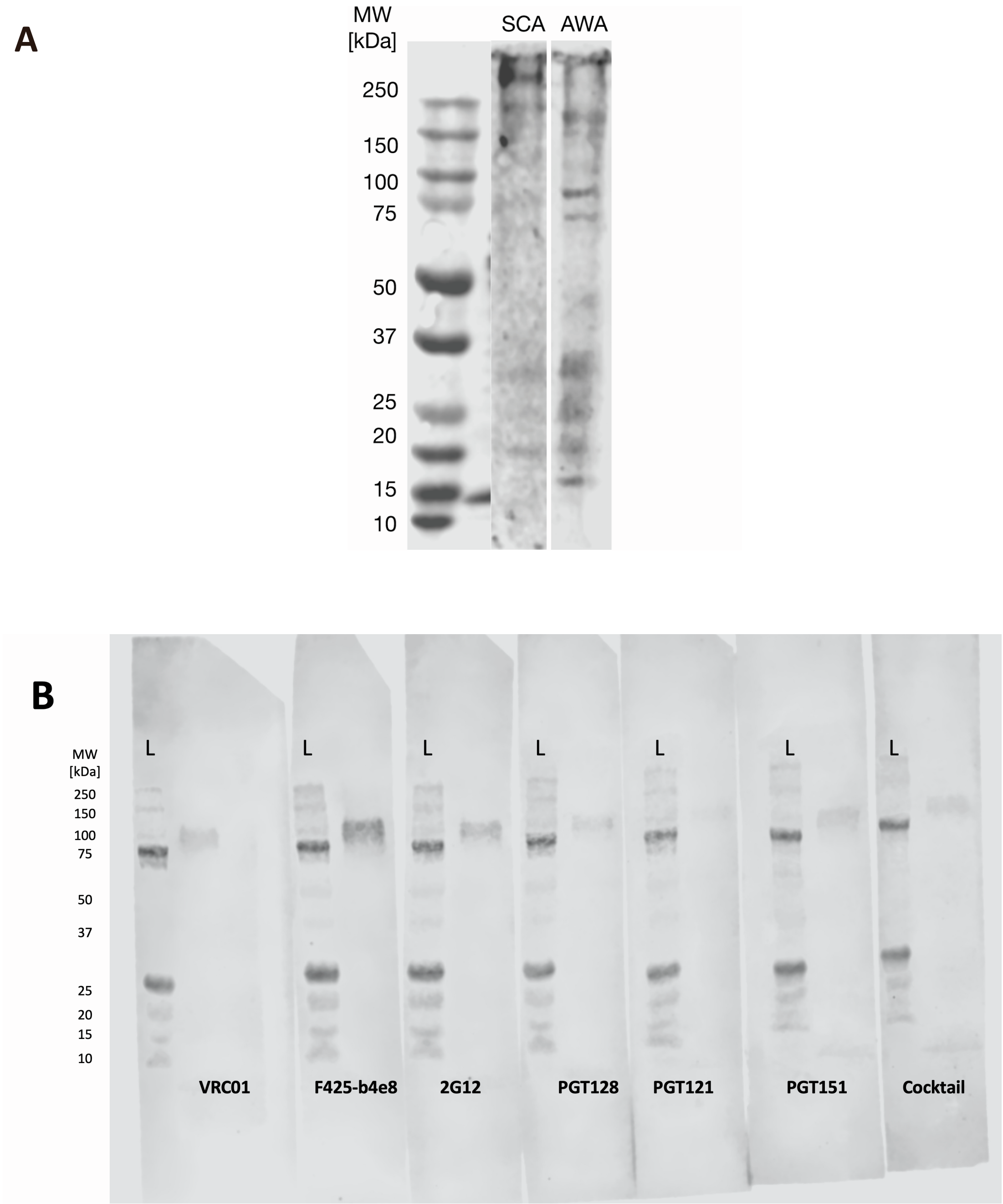

Figure S4: Western blot of SCA/AWA antigens with sera from *S. mansoni* infected individuals and gp120 with HIV-1 bnAbs. A) Binding of sera from *S. mansoni* infected individuals to SCA and AWA by western blot. **B)** Binding to HIV-1 bnAbs (VRC01, F425-b4e8, 2G12, PGT128, PGT121, PGT151 or a cocktail of all mAbs) to recombinant gp120 (JR-FL) by western blot. A denatured, non-reducing gel is used. PGT121, PGT128, PGT151 and 2G12 are all glycan reactive bnAbs. L = ladder. This figure relates to **Figure 3A.**

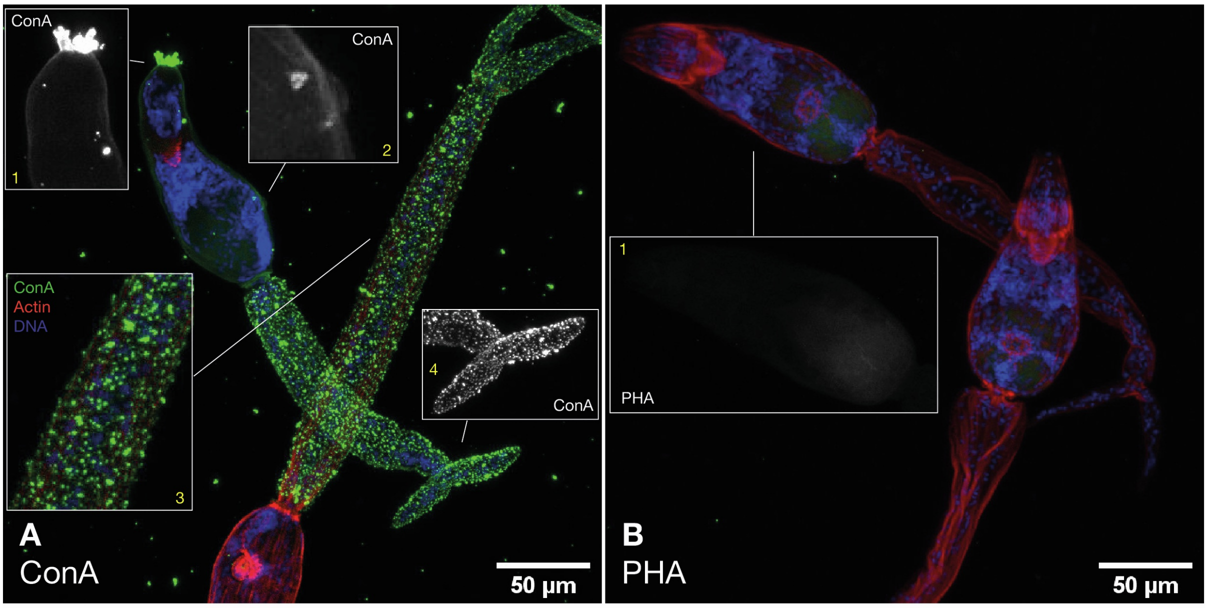

Figure S5: Confocal microscopy images of *S. mansoni* cercariae stained with lectins ConA and PHA-L. Lectins were detected with AlexaFluor488 (green), actin was detected with rhodamine (red) and DNA was stained with DAPI (blue). Black and white images show projections of lectin staining only, while coloured images show overlays. **A)** Confocal microscopy of *S. mansoni* cercariae with ConA, a mannose and glucose binding lectin, which recognizes high-mannose glycans, and some hybrid *N*-glycans. ConA binds to the duct opening on the oral sucker (**1**) and the ventral sucker (**2**). ConA also recognizes surface motifs (spines, ciliated sensory papillae and basement membrane/tegument/glycocalyx structures) above the actin-layer (**3**), and ciliated sensory papillae and basement membrane/tegument/glycocalyx structures (**4**). **B**) Confocal microscopy of *S. mansoni* cercariae with Phytohaemagglutinin-L (PHA-L), a lectin recognizing galactose within tri- and tetra-antennary, complex-type *N*-glycan structures. PHA-L only weakly binds the pre- and post-scetabular gland system (**1**), which is sits interior of the parasite. Confocal microscopy staining was performed twice on different cercariae preparations. Representative images are shown from one experiment. This figure relates to **Figure 3C-F**.

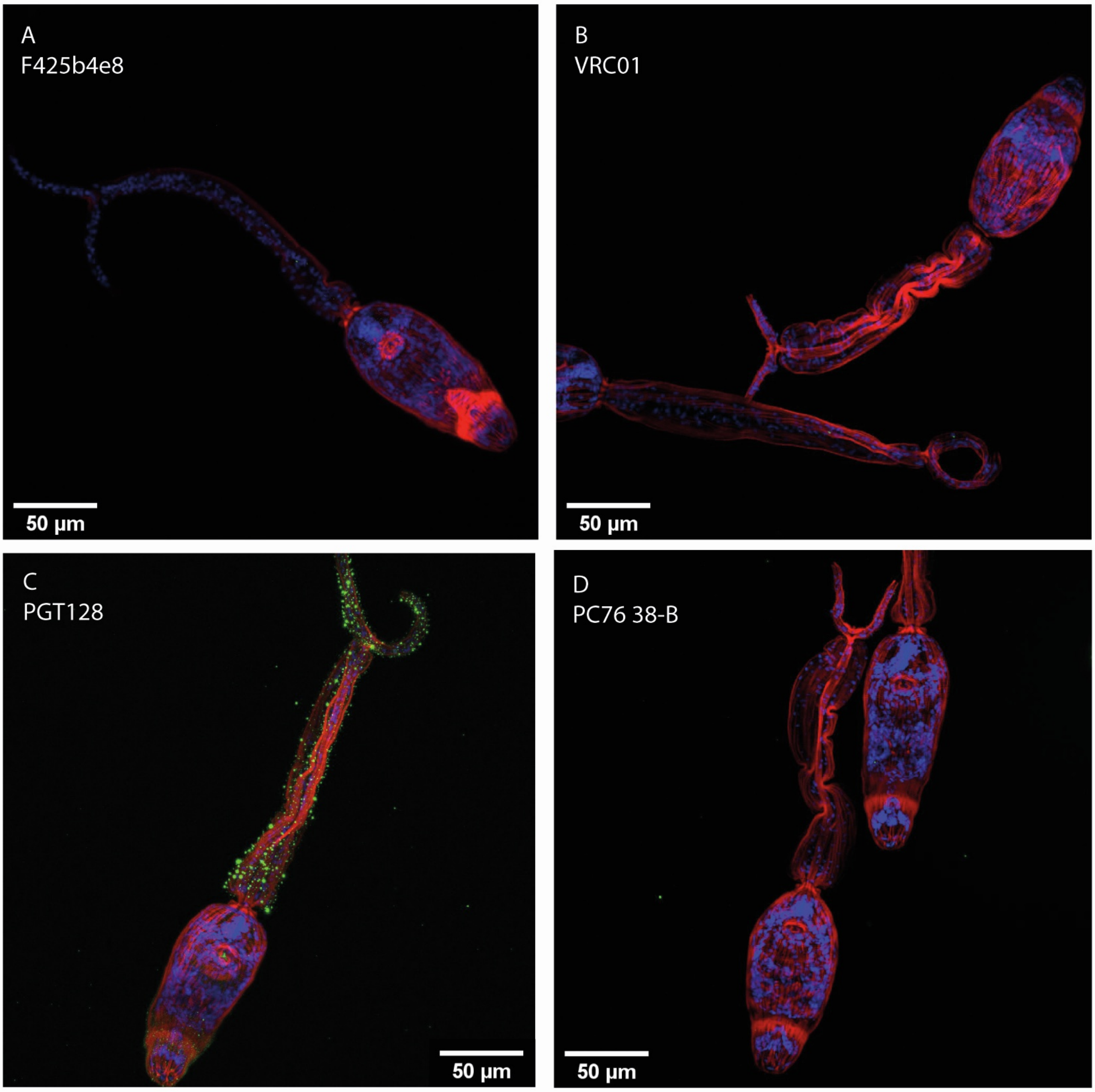

Figure S6: Confocal microscopy images of *S. mansoni* cercariae with control bnAbs and bnAbs not displaying cross-reactivity. Glycan independent HIV-1 bnAbs F425-b4e8 (**A**) and VRC01 (**B**) were used as negative controls. HIV-1 bnAb PGT128 cross-reactivity *S. mansoni* cercariae was determined previously and was included when staining PCDN76 bnAb lineage as a positive control (**C**). PCDN76 clonal variant, 38-B, did not display any cross-reactivity with *S. mansoni* cercariae (**D**). HIV-1 bnAbs were detected with AlexaFluor488 (green), actin was detected with rhodamine (red) and DNA was stained with DAPI (blue). Confocal microscopy staining was performed twice on different cercariae preparations. Representative images are shown from one experiment. This figure relates to **Figures 3C-F** and **Figures 5C-H**.

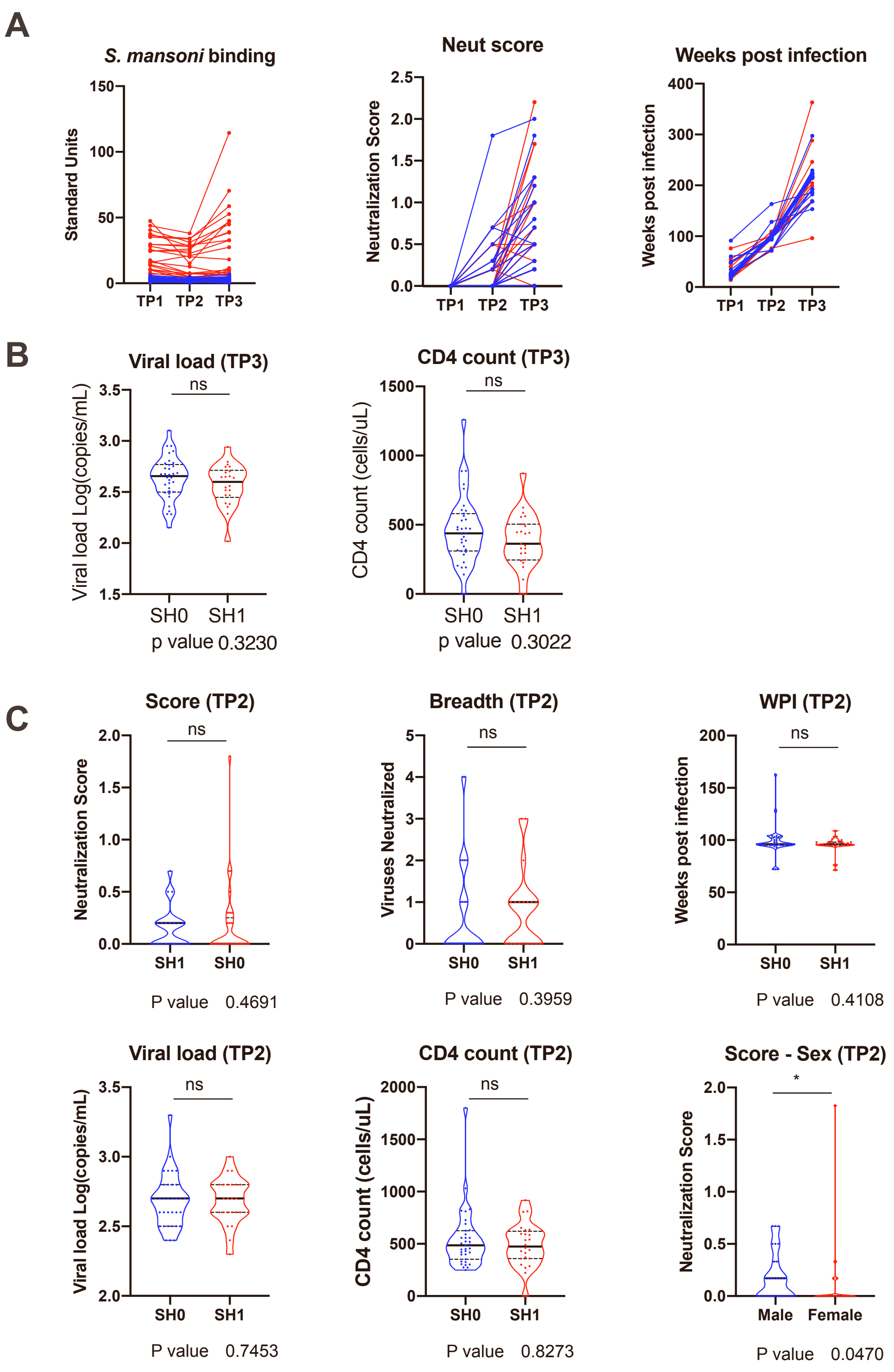

Figure S7: Analysis of HIV-1 neutralization and *S. mansoni* seroprevalence in IAVI Protocol C donors. **A)** *S. mansoni* binding, neutralization score and weeks post infection (WPI) at TP1, TP2 and TP3. SH1 donors are shown in red and SH0 donors are shown in blue. Donors were considered *S. mansoni* seropositive if the SEA binding was above 6.8 Units. **B)** Comparison of CD4 count and viral load at TP3 between donors with *S.*

mansoni seroreactivity (SH1, red) and no *S. mansoni* seroreactivity (SH0, blue). P-values were calculated using Mann-Whitney U tests and significances were assigned as follows: not significant (ns), <0.0332 (*), <0.0021 (**), <0.0002 (***) and <0.00001 (****). **C)** Comparison of neutralization score, the number of viruses neutralized (breadth), weeks post infection, viral load, CD4 count and sex between donors with *S. mansoni* seroreactivity (SH1) and no *S. mansoni* seroreactivity (SH0) at TP2. Statistical differences were determined using Mann-Whitney tests. P-values were reported as follows: not significant (ns), <0.0332 (*), <0.0021 (**), <0.0002 (***) and <0.00001 (****). This figure relates to **Figure 4**.

^{13}C δ	^{1}H δ	Ring identity	Proton identity
**27.756**	1.078	Pentylamine	Hc/Hc'
**27.756**	1.274	Pentylamine	Hd/Hd'
**26.169**	1.346	Pentylamine	Hb/Hb'
**21.763**	1.711	β-GlcNAc6	CH3
**21.763**	1.723	β-GlcNAc2	CH3
**21.763**	1.748	β-GalNAc1	CH3
**21.763**	1.759	β-GlcNAc5	CH3
**38.955**	2.653	Pentylamine	Ha/Ha'
**64.599**	2.93	β-Xyl	H5/H5'
**72.953**	3.054	β-Xyl	H2
**74.899**	3.11	β-Xyl	H3
**66.765**	3.177	Man 3	H4
**74.209**	3.177	β-GlcNAc2	H3
**74.209**	3.18	β-GlcNAc6	H3
**69.83**	3.263	Chain	He/He'
**61.535**	3.28	β-GlcNAc6	H6/H6'
**78.701**	3.301	β-GlcNAc6	H4
**68.951**	3.31	β-Xyl	H4
**74.059**	3.314	β-GlcNAc5	H5
**72.389**	3.322	α-Man 4'	H3?
**66.311**	3.331	β-GlcNAc 5(/b-GalNAc 1)	H6
**78.664**	3.333	β-GlcNAc2	H4
**59.71**	3.349	β-GlcNAc2	H6
**71.983**	3.352	β-Man 4	H4/H5?
**74.059**	3.354	β-GalNAc1	H5
**54.676**	3.391	β-GlcNAc6	H2
**69.303**	3.395	α-Man 4'	H5
**54.237**	3.401	β-GlcNAc 2	H2
**71.671**	3.402	β-Man 4	H4/H5?
**75.032**	3.402	β-GalNAc 1	H4
**59.525**	3.422	β-GlcNAc 5/b-GalNAc 1	H6
**70.32**	3.426	β-GlcNAc 5/b-GalNAc 1	H3
**71.633**	3.426	β-GlcNAc 5/b-GalNAc 1	H3
**79.071**	3.427	β-GlcNAc 5	H4
**64.895**	3.446	β-Man 4	H6/H6'
**60.619**	3.447	α-Man 3	H6/H6'
**54.66**	3.47	β-GlcNAc 5	H2
**60.677**	3.476	β-Man 4	H6
**68.997**	3.529	α-Man 3	H3
**59.71**	3.533	β-GlcNAc2	H6'
**70.146**	3.537	α-Man 4'	H4?
**59.525**	3.547	β-GlcNAc 5(/b-GalNAc 1)	H6
**76.563**	3.564	β-Man 4	H3

**60.619**	3.564	α-Man 3	H6/H6'
**73.347**	3.573	α-Man 3	H5
**69.83**	3.587	Pentylamine	He/He'
**61.535**	3.602	β-GlcNAc6	H6/H6'
**52.162**	3.608	β-GalNAc 1	H2
**67.218**	3.614	β-GlcNAc 2	H5
**64.895**	3.65	β-Man 4	H6/H6'
**69.434**	3.663	α-Man 4'	H2
**64.599**	3.695	β-Xyl	H5/H5'
**76.037**	3.826	α-Man 3	H2
**79.121**	3.94	β-Man 4	H2
**104.929**	4.107	β-Xyl	H1
**100.761**	4.167	β-GlcNAc6	H1
**101.424**	4.185	β-GalNAc 1	H1
**99.282**	4.192	β-GlcNAc 2	H1
**101.021**	4.273	β-GlcNAc 5	H1
**100.236**	4.561	β-Man 4	H1
**99.272**	4.596	α-Man 4'	H1
**98.695**	4.814	α-Man 3	H1

Supplemental Table 1: Glycan 28 ^{1}H and ^{13}C NMR assignment. This table relates to **Figure 2**.

PC Code	Sex	Age at infection	Overall SH Status	Best Neut Score	Score Category	Overall Specificity Mapping	SH sero-reactivity 1	WPI 1	Virus Breadth 2	Neut Score (TP2)	SH sero-reactivity 2	WPI 2	Virus breadth 3	Neut Score (TP3)	SH sero-reactivity 3	WPI 3	ID50 TP3 (Geomean)
PC092	m	30	0	2.2	TOP	Mixed	3.8	24	1	0.17	2.8	96	5	1.83	3.9	218	204
PC064	f	29	0	2.0	TOP	Quaternary Apex	1.4	19	4	1.83	0.3	128	5	2.00	1.4	153	245
PC094	f	29	0	1.7	TOP	Glycan Supersite	3.2	48			1.6	96	4	1.33	2.1	216	97
PC022	f	29	0	1.5	TOP	Glycan Supersite	2.6	91	2	0.33	1.7	163	4	1.33	2.2	186	97
PC035	m	49	0	1.5	TOP	Quaternary Apex	5.3	23	1	0.17	3.0	95	3	1.00	2.3	216	55
PC036	m	24	0	1.3	TOP	Glycan Supersite	0.9	24	2	0.67	1.0	98	5	1.33	0.8	214	118
PC041	f	41	0	1.2	TOP	Interface	4.4	20	0	0.00	2.5	104	4	1.17	2.7	297	80
PC048	f	38	0	1.2	TOP	Interface	3.9	17			0.3	100	5	1.17	2.3	223	98
PC258	f	23	0	1.2	TOP	Mixed	2.4	25			1.3	72	4	1.17	1.3	168	80
PC037	m	20	0	1.2	TOP	Glycan Supersite	3.9	23	1	0.33	4.8	104	3	0.83	6.5	182	102
PC181	m	41	0	1.2	TOP	Glycan Supersite	2.0	24	2	0.50	1.3	96	3	1.00	1.1	168	55
PC082	m	27	0	1.2	TOP	Glycan Supersite	1.5	24	4	0.67	1.6	96	3	0.50	2.5	192	41
PC067	m	23	0	1.0	TOP	Glycan Supersite	5.2	19	1	0.17	2.1	103	2	0.50	1.5	225	26
PC003	m	22	0	0.8	MED	Glycan Supersite	3.4	25			1.8	72	2	0.33	0.5	222	48
PC050	m	24	0	0.8	MED	Interface	2.0	50	2	0.33	2.0	98	4	0.83	0.6	216	73
PC178	f	19	0	0.8	MED	Interface	0.7	24			1.3	96	1	0.33	4.3	191	18
PC049	f	22	0	0.7	MED	Quaternary Kif-	1.6	25	0	0.00	2.1	95	3	0.67	1.8	214	65
PC163	m	45	0	0.7	MED		2.5	23	0	0.00	3.7	96	1	0.33	5.0	216	18
PC144	f	25	0	0.7	MED	CD4bs?	2.0	23	0	0.00	1.3	96	4	0.67	0.0	216	46
PC074	f	20	0	0.5	MED		2.0	60			3.4	72	3	0.50	3.4	193	54
PC034	m	26	0	0.5	MED		1.9	19	0	0.00	1.5	102	1	0.17	2.9	223	19
PC089	m	27	0	0.5	MED		3.2	16	0	0.00	2.5	104	2	0.50	2.9	224	26
PC088	f	26	0	0.3	WEAK		1.9	23	0	0.00	3.1	95	0	0.00	1.7	218	17
PC102	f	31	0	0.3	WEAK		1.2	24	0	0.00	1.0	96	1	0.17	0.2	216	25
PC047	f	25	0	0.3	WEAK		2.7	25	0	0.00	1.0	95	1	0.33	0.7	216	18
PC090	f	46	0	0.3	WEAK		1.5	24	0	0.00	2.1	96	1	0.17	2.7	216	19
PC093	f	42	0	0.3	WEAK		2.7	19	0	0.00	2.0	103	1	0.17	2.0	223	25
PC057	f	21	0	0.2	WEAK		4.2	22	0	0.00	2.8	96	0	0.00	2.9	219	10
PC286	f	28	0	0.2	WEAK		5.9	27	0	0.00	4.2	96	0	0.00	3.4	216	10
PC009	m	33	0	0.2	WEAK		3.0	28	0	0.00	2.1	100	0	0.00	1.7	216	10
PC027	m	24	0	0.2	WEAK		0.8	34	0	0.00	0.7	93	0	0.00	0.8	229	10
PC327	m	39	0	0.2	WEAK		2.2	18	0	0.00	1.5	104	1	0.17	1.4	223	33
PC141	m	50	0	0.2	WEAK		3.2	24	0	0.00	3.0	95	0	0.00	4.3	215	17
PC147	m	24	0	0.0	WEAK		1.1	24	0	0.00	1.0	96	0	0.00	3.2	216	10
PC059	m	32	0	0.0	WEAK		2.1	24	0	0.00	0.6	94	0	0.00	2.9	215	10
PC068	m	33	1	2.3	TOP	Quaternary Kif+	16.0	21	1	0.17	12.7	95	5	2.17	7.2	214	295
PC039	m	27	1	2.0	TOP	Glycan Supersite	7.8	23			4.4	71	5	1.67	5.0	214	223
PC023	m	34	1	1.8	TOP	Interface	4.0	23	0	0.00	2.3	95	5	1.67	11.6	288	170
PC268	m	33	1	1.7	TOP	Glycan Supersite	2.3	15	1	0.17	0.0	101	4	1.67	10.2	169	238
PC174	f	23	1	1.3	TOP	Quaternary Apex	15.8	40	0	0.00	21.0	109	4	1.33	27.4	204	97
PC002	m	22	1	1.2	TOP	Quaternary Kif+	29.7	14	3	0.67	27.9	98	4	1.00	39.1	169	88
PC030	m	44	1	1.2	TOP	Glycan Supersite	24.5	55			24.5	103	5	1.17	70.4	246	98
PC076	m	28	1	1.2	TOP	Glycan Supersite	29.6	47	1	0.17	29.1	95	4	1.00	32.8	216	88
PC029	f	37	1	1.2	TOP	Glycan Supersite	13.6	47	0	0.00	5.0	96	6	1.17	4.3	192	120
PC063	f	34	1	1.0	TOP	CD4bs	10.4	24	0	0.00	5.0	96	4	1.00	2.8	363	88
PC115	f	21	1	0.8	MED		35.2	24	0	0.00	33.7	97	2	0.67	58.7	217	31
PC016	m	28	1	0.8	MED	Quaternary Kif+	40.6	26			31.2	96	2	0.50	44.8	199	44
PC031	m	28	1	0.8	MED	MPER	25.5	24	1	0.17	20.2	98	3	0.67	18.2	216	38
PC043	f	26	1	0.8	MED	Glycan Supersite	14.2	14	1	0.17	7.7	95	3	0.50	10.3	216	71
PC077	m	52	1	0.7	MED	Quaternary Kif-	6.9	76	0	0.00	7.3	100	0	0.00	8.6	217	10
PC079	f	34	1	0.7	MED		36.3	16			34.0	76	2	0.50	52.7	96	34
PC010	m	46	1	0.7	MED		10.6	25	1	0.17	7.3	98	2	0.50	5.9	215	34
PC097	m	29	1	0.7	MED	Quaternary Apex	10.0	47	3	0.50	2.5	96	2	0.33	2.5	215	28
PC018	m	25	1	0.5	MED		43.8	25			38.1	95	1	0.17	114.4	216	33
PC069	m	28	1	0.5	MED	Quaternary Apex	37.6	24	0	0.00	28.3	99	3	0.50	38.6	216	32
PC236	m	26	1	0.5	MED		17.6	23	2	0.50	7.3	95	3	0.50	6.9	217	41
PC101	m	25	1	0.3	WEAK		28.2	22	0	0.00	22.7	94	1	0.17	32.7	216	15
PC038	m	45	1	0.2	WEAK		24.5	24	0	0.00	26.1	96	0	0.00	46.9	216	13
PC107	f	25	1	0.2	WEAK		47.4	24	1	0.17	15.2	95	0	0.00	47.6	222	10

Supplemental Table 2: IAVI Protocol C cohort donors

The PC code represents the donor identifier and sex is reported with 'm' for male and 'f' for female. Clinical data, neutralization capacity and bnAb specificity was previously determined and reported by Elise Landais and Pascal Poingard and co-workers (Landais et al, 2016). The best overall neutralization score is reported and categorized by 'TOP' for top neutralizers (score ≥1), 'MED' for medium neutralizers (score <1 and ≥0.5) and 'WEAK' for weak neutralizers (score <0.5). Neutralization breadth is reported as number of viruses neutralized out of a 6-virus panel (Landais et al, 2016). Neutralization score (Score) is a numerical representation of neutralization potency of breadth. Geometric mean of ID_{50} as well as *S. mansoni* seroreactivity in standard units and weeks post infection (WPI) are reported for three time points marked as 'TP1','TP2' and 'TP3'. Donor PC076 is highlighted in red. This table relates to **Figure 4**.
