# Supplementary material for: Cross-reactivity of glycan-reactive HIV-1 broadly neutralizing antibodies with parasite glycans
Source: Cell Rep. 2022 Mar 29;38(13):110611. doi: 10.1016/j.celrep.2022.110611 (PMC10073069; doi:10.1016/j.celrep.2022.110611)
Supplement: Document S3. Article plus supplemental information [file mmc3.pdf]

Cell Reports

Article

Cross-reactivity of glycan-reactive HIV-1 broadly neutralizing antibodies with parasite glycans

Graphical abstract

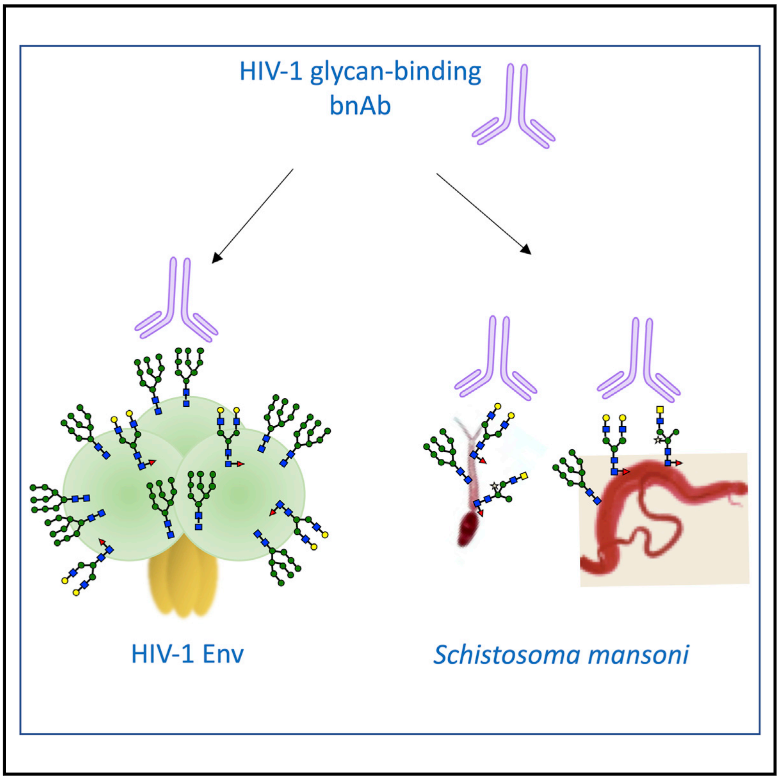

Highlights

- Glycan-binding HIV-1 bnAbs cross-react with parasite self- and non-self *N*-glycans
- These bnAbs also bind glycoprotein antigens from different life stages of *S. mansoni*
- Unmutated precursor of an N332/V3 lineage binds *S. mansoni* antigens but not gp120

Authors

Isabella Huettner, Stefanie A. Krumm, Sonia Serna, ..., Jesus Angulo, Niels Reichardt, Katie J. Doores

Correspondence

katie.doores@kcl.ac.uk

In brief

Huettner et al. show that glycan-binding HIV-1 broadly neutralizing antibodies (bnAbs) and the unmutated common ancestor of an N332/V3 bnAb lineage cross-react with self- and non-self *N*-glycans and glycoprotein antigens from the different life stages of *Schistosoma mansoni*. This presents a potential strategy for elicitation of glycan-reactive bnAbs through vaccination.

Huettner et al., 2022, Cell Reports *38*, 110611
March 29, 2022 © 2022 The Author(s).
https://doi.org/10.1016/j.celrep.2022.110611

CellPress

Cell Reports

CellPress
OPEN ACCESS

Article

Cross-reactivity of glycan-reactive HIV-1 broadly neutralizing antibodies with parasite glycans

Isabella Huettner,[1] Stefanie A. Krumm,[1] Sonia Serna,[2] Katarzyna Brzezicka,[2] Serena Monaco,[3] Samuel Walpole,[3] Angela van Diepen,[4] Fiona Allan,[5] Thomas Hicks,[3] Simon Kimuda,[1] Aidan M. Emery,[5] The IAVI Protocol C Investigators & The IAVI African HIV Research Network,[10] Elise Landais,[6,7] Cornelis H. Hokke,[4] Jesus Angulo,[3,8] Niels Reichardt,[2,9] and Katie J. Doores[1,11,*]

[1]Department of Infectious Diseases, School of Immunology & Microbial Sciences, King's College London, London, UK
[2]Glycotechnology Laboratory, Center for Cooperative Research in Biomaterials (CIC biomaGUNE), Basque Research and Technology Alliance (BRTA), Paseo Miramón 182, 20014 San Sebastian, Spain
[3]School of Pharmacy, University of East Anglia, Norwich Research Park, Norwich, Norfolk NR4 7TJ, UK
[4]Department of Parasitology, Leiden University Medical Center, Leiden, the Netherlands
[5]Department of Life Sciences, Natural History Museum, Cromwell Road, London, UK
[6]International AIDS Vaccine Initiative Neutralizing Antibody Center, La Jolla, CA 92037, USA
[7]International AIDS Vaccine Initiative, New York, NY 10004, USA
[8]Instituto de Investigaciones Químicas (CSIC-US), Avda. Américo Vespucio, 49, 41092 Sevilla, Spain
[9]CIBER-BBN, Paseo Miramón 182, 20009 San Sebastian, Spain
[10]List of investigators and details of affiliations are listed in the acknowledgements
[11]Lead contact
*Correspondence: katie.doores@kcl.ac.uk
https://doi.org/10.1016/j.celrep.2022.110611

SUMMARY

The HIV-1 Envelope glycoprotein (Env) is the sole target for broadly neutralizing antibodies (bnAbs). Env is heavily glycosylated with host-derived *N*-glycans, and many bnAbs bind to, or are dependent upon, Env glycans for neutralization. Although glycan-binding bnAbs are frequently detected in HIV-infected individuals, attempts to elicit them have been unsuccessful because of the poor immunogenicity of Env *N*-glycans. Here, we report cross-reactivity of glycan-binding bnAbs with self- and non-self *N*-glycans and glycoprotein antigens from different life-stages of *Schistosoma mansoni*. Using the IAVI Protocol C HIV infection cohort, we examine the relationship between *S. mansoni* seropositivity and development of bnAbs targeting glycan-dependent epitopes. We show that the unmutated common ancestor of the N332/V3-specific bnAb lineage PCDN76, isolated from an HIV-infected donor with *S. mansoni* seropositivity, binds to *S. mansoni* cercariae while lacking reactivity to gp120. Overall, these results present a strategy for elicitation of glycan-reactive bnAbs which could be exploited in HIV-1 vaccine development.

INTRODUCTION

Elicitation of broadly neutralizing antibodies (bnAbs) against HIV-1 is thought to be one of the key steps toward the development of an effective HIV-1 vaccine. bnAbs arise in 10%–30% of HIV-1-infected individuals after 2–3 years of infection and can neutralize a broad range of HIV-1 strains through binding to conserved regions on the HIV-1 surface glycoprotein, Envelope (Env) (Gray et al., 2011; Hraber et al., 2014; Simek et al., 2009). Env consists of a trimer of gp120 and gp41 heterodimers that is heavily glycosylated with host-derived *N*-linked glycans. The dense clustering of *N*-glycans on Env sterically limits their accessibility to glycan-processing enzymes, leading to an abundance of under-processed, oligomannose-type glycans that form a non-self-motif termed the "mannose-patch" (Behrens et al., 2016; Doores et al., 2010a; Pritchard et al., 2015; Zhu et al., 2000). It was originally thought that the extensive glycosylation on Env shielded conserved regions of the protein from the immune system, but recent studies have revealed that this "glycan shield" can also be the target of some of the most broad and potent HIV-1 bnAbs isolated from infected individuals. Conserved bnAb epitopes that incorporate Env *N*-glycans include the N332/V3 epitope (targeted by representative bnAbs PGT128, PGT121, PGT135, BG18, and DH270.6) (Bonsignori et al., 2017; Freund et al., 2017; Sok et al., 2016; Walker et al., 2011), the N160/V2 epitope (targeted by representative bnAbs PGT145, PG9, and CAP256-VRC26) (Burton et al., 2009; Doria-Rose et al., 2014; Walker et al., 2011), and an epitope at the gp120/gp41 interface (targeted by representative bnAbs PGT151 and 35O22) (Falkowska et al., 2014; Huang et al., 2012).

Elicitation of bnAbs against these glycan-dependent epitopes is highly desirable in a vaccine setting because of their high neutralization breadth and their ability to protect at low serum concentrations in simian-human immunodeficiency virus (SHIV) challenge models (Mascola et al., 1999; Moldt et al., 2012). Yet although anti-glycan bnAbs are elicited during natural

This is an open access article under the CC BY license (http://creativecommons.org/licenses/by/4.0/).

infection, attempts to re-elicit them through vaccination have thus far been largely unsuccessful (Dubrovskaya et al., 2019; Mccoy et al., 2016; Pauthner et al., 2019; Ringe et al., 2018; Sanders et al., 2015; Wagh et al., 2018). Immunization with state-of-the-art immunogens, which resemble Env as displayed on the virion surface, has not led to glycan-binding bnAbs or Abs (Ringe et al., 2018) but has instead induced strain-specific autologous neutralizing antibodies (nAbs) targeting protein residues within "holes" in the Env glycan shield rather than the glycans themselves (Mccoy et al., 2016). A better understanding of how glycan-reactive bnAbs arise during natural HIV-1 infection will be important for development of immunogens aimed at eliciting bnAbs against Env *N*-glycans.

Some pathogens display non-mammalian (non-self) glycans that are highly immunogenic and that generate a robust anti-glycan antibody (Ab) response (Astronomo and Burton, 2010; van Diepen et al., 2012; Jones, 2005; Yang et al., 2018). These glycans form the basis of established polysaccharide conjugate vaccines that protect against *Streptococcus pneumoniae*, *Haemophilus influenzae type b* (Hib), and *Neisseria meningitidis* infections (Astronomo and Burton, 2010; Jones, 2005; MacCalman et al., 2020; Mettu et al., 2020). In contrast, some pathogens use mammalian self-glycans to evade the host immune system, and these are typically poorly immunogenic and pose a greater challenge for vaccine development. First-generation HIV-1 bnAb 2G12, a bnAb with modest breadth and potency that binds the terminal Manα1,2Man residues of $Man_9GlcNAc_2$ D1 arm, has previously been shown to cross-react with Manα1,2Man motifs on *Candida albicans* (Doores et al., 2010b; Dunlop et al., 2008), with influenza haemagglutinin (Lee et al., 2021) and with a lipopolysaccharide (LPS) from plant bacteria *Rhizobium radiobacter* Rv3 (Clark et al., 2012). Glycan-reactive HIV-1 nAbs isolated from simian-human immunodeficiency virus-infected macaques have also been shown to cross-react with other glycosylated pathogens, including *Candida albicans*, and with the severe acute respiratory syndrome coronavirus 2 (SARS-CoV-2) Spike protein (Williams et al., 2021). Furthermore, a recent study by Wardemann and co-workers also demonstrated that glycan-binding human monoclonal Abs (mAbs) isolated against *Klebsiella pneumoniae* efficiently neutralize *Pseudomonas luteola* lipopolysaccharide *in vitro* and bind HIV-1 Env (Rollenske et al., 2018). These observations lead us to hypothesize that development of HIV-1 glycan-reactive bnAbs could be guided by pre-existing glycan-reactive B cells generated by infections with other highly glycosylated pathogens displaying immunogenic non-self glycans or self-glycans in a non-self context (modified-self). In support of this hypothesis, Haynes and co-workers showed that HIV-1 Envs from early transmitted/founder viruses bind to pre-existing B cells generated against non-HIV-1 protein antigens on gut microflora, suggesting a role for cross-pathogen or cross-antigen priming in HIV-1 antibody development against protein antigens (Liao et al., 2011; Trama et al., 2014; Williams et al., 2015).

Here we investigated the cross-reactivity of broad and potent second-generation glycan-reactive HIV-1 bnAbs (including PGT121, PGT128, PGT151), with self and non-self glycan structures found on other glycosylated pathogens and explore the role cross-pathogen priming might play in bnAb development using plasma from the IAVI Protocol C HIV-1 infection cohort (Landais et al., 2016). We show that glycan-binding HIV-1 bnAbs bind to defined glycans (mammalian self and non-self) present on the different life stages of *Schistosoma mansoni*, as well as to native glycoproteins solubilized from the cercariae, adult worm, and egg life stages. Furthermore, we demonstrate binding of several bnAbs to intact *S. mansoni* cercariae by confocal microscopy. Using the IAVI protocol C HIV-1 infection cohort (Landais et al., 2016), we examined the relationship between *S. mansoni* seropositivity and development of HIV-1 neutralizing activity targeted against glycan-dependent epitopes. Finally, we show that the unmutated common ancestor (UCA) of the N332/V3 PCDN76 bnAb lineage, isolated from an HIV-1 infected donor with *S. mansoni* seropositivity, was found to bind to *S. mansoni* cercariae while lacking reactivity to recombinant gp120 in ELISA. These data suggest that parasite glycans could have played a role in priming the bnAb responses in donor PC076 and present a strategy, which could be exploited in HIV-1 vaccine development.

RESULTS

Binding of HIV-1 glycan-reactive bnAbs to a synthetic parasite glycan microarray

To address the hypothesis that cross-pathogen priming could play a role in development of glycan-reactive HIV-1 bnAbs, we first measured binding of glycan-reactive bnAbs PGT121, PGT123, PGT128, PGT130, PGT151, and a CD4-binding site bnAb, PGV04, to a synthetic glycan microarray with a focus on helminth and plant *N*-glycan structures (Brzezicka et al., 2015; Echeverria et al., 2018). The glycan array displayed chemically synthesized *N*-glycans found on the blood trematode *S. mansoni* and, because of the stepwise chemo-enzymatic buildup of glycan structures, also included *N*-glycan fragments not typically found in nature (Figure S1). We observed binding of N332/V3 bnAb PGT121, and somatic variant PGT123, to mammalian glycans including bi-, tri-, and tetra-antennary complex glycans (**59**–**62**) as well as non-mammalian, non-self bi-antennary complex-glycan structures (**24**, **25**, **28**, **29**, **37**, **63**, and **80**) displaying terminal LacDiNAc motifs and/or core xylose and *bis*-core fucosylation (Figure 1A). The pattern of PGT121 glycan binding suggests that the minimal glycan structure required for binding is the core pentasaccharide ($Man_3GlcNAc_2$) as well as a complete α3 arm. However, modifications to the non-reducing end of this arm and to the glycan core are tolerated in binding. Gp41/gp120 interface bnAb, PGT151, bound to self tri- and tetra-antennary complex-glycans (**60** and **62**), as previously observed (Falkowska et al., 2014) (Figure 1A). No binding was observed for N332/V3 bnAbs PGT128 and PGT130 (data not shown), which have previously been shown to bind to high-mannose glycans that are not present on this synthetic glycan microarray (Doores et al., 2015; Walker et al., 2011). No binding was observed for control bnAb PGV04 (data not shown) (Falkowska et al., 2012). Therefore, HIV-1 glycan-binding bnAbs bind glycans found in the glycome of the *S. mansoni* parasite.

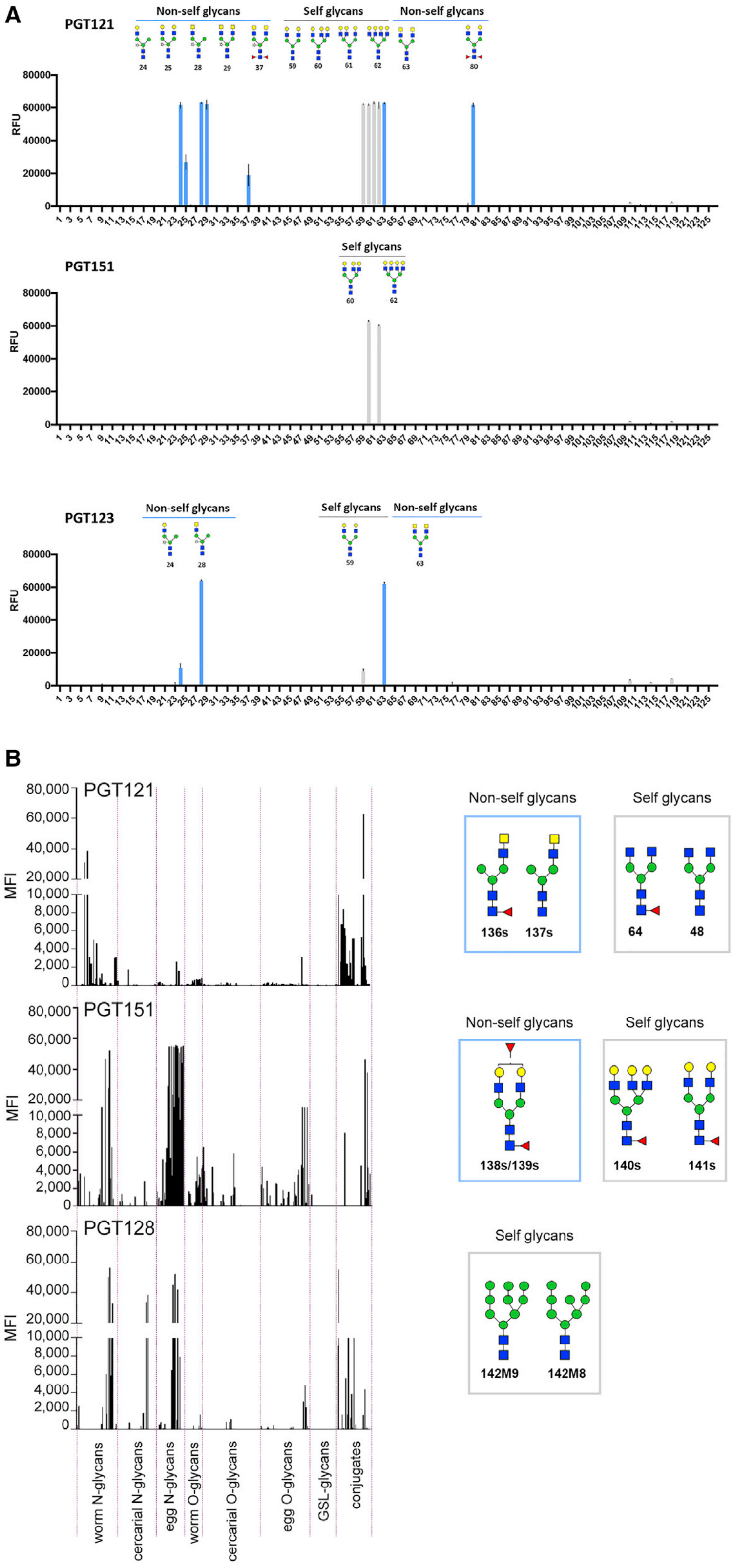

Figure 1. Binding of HIV-1 bnAbs to *S. mansoni* parasite-derived glycans on glycan microarrays

(A) Synthetic glycan microarray displaying *S. mansoni*-derived *N*-glycans and fragment structures as previously described (Brzezicka et al., 2015; Echeverria et al., 2018). No high-mannose glycan structures are present on this array. Glycans bound by the HIV-1 bnAbs PGT121, PGT151, and PGT123 are displayed. Mammalian "self" and "non-self" glycans are shown in gray and blue, respectively. Binding is reported in relative fluorescence units (RFU). Each histogram represents the average RFU values for four replicates spots and the error bars represent the SD of the mean. A full list of glycan structures present on the synthetic glycan array can be found in Figure S1.

(B) Shotgun glycan microarray of *S. mansoni*-extracted glycans as previously described (De Boer et al., 2007; van Diepen et al., 2012, 2015). Binding of HIV-1 bnAbs PGT121, PGT151, and PGT128 determined. Each line represents the average mean fluorescence intensity (MFI) values for three replicates spots. Glycans bound are displayed on the right and mammalian "self" glycans and non-mammalian "non-self" glycan motifs are indicated. The life stage and type of glycan (*N*-, *O*-, or glycosphingolipid [GSL] glycan) are reported. Glycan structures with "s" indicate structures only found on the shotgun glycan microarray. Man, green circle; GlcNAc, blue square; GalNAc, yellow square; Gal, yellow circle; sialic acid, purple diamond; fucose, red triangle; xylose, star.

Binding of selected HIV-1 glycan-reactive bnAbs to a *S. mansoni* shotgun glycan microarray

We next used a shotgun glycan microarray displaying *N*- and *O*-glycans and glycolipid glycans isolated from different life stages of *S. mansoni* (cercariae, worm and egg) to further assess cross-reactivity between HIV-1 glycan-reactive bnAbs PGT121, PGT151, and PGT128 and *S. mansoni* glycans and life stages. Unlike the synthetic glycan microarray, the shotgun array also included high-mannose glycans, which are known targets for several highly potent glycan-binding HIV-1 bnAbs, including PGT128 (Bonsignori et al., 2017; Walker et al., 2011). Each glycan fraction contained one or more glycan structures that were assigned by mass spectrometry (De Boer et al., 2007; van Diepen et al., 2012, 2015). PGT128 bound $Man_8GlcNAc_2$ (**142M8**) and $Man_9GlcNAc_2$ (**143M9**) isolated from the worm, cercariae, and egg life stages (Figure 1B); PGT121 bound to mono-antennary glycans **136s** and **137s** displaying a terminal LacDiNAc motif and non-galactosylated bi-antennary glycans **48** and **64** also found in worms, cercariae and eggs (Figure 1B). PGT151 bound bi- and tri-antennary self-glycans **140s**–**141s** and terminal fucosylated non-self bi-antennary glycans **138s**–**139s** isolated from worms and eggs (Figure 1B). Therefore, selected glycan-reactive HIV-1 bnAbs bind *N*-glycans expressed by the life stages of *S. mansoni* that humans are exposed to during infection.

PGT121 binds non-self bi-antennary *N*-glycans in a similar mode to self *N*-glycans

PGT121 binds two *N*-linked glycans on HIV Env, the high-mannose glycan at N332 and a complex *N*-glycan from the V1 loop, as well as interacting with the V3 loop (Julien et al., 2013a). To determine whether the cross-reactivity of PGT121 to non-self parasite-derived glycans is mediated by similar Fab interactions as those already reported for the self complex-type *N*-glycan **144SIA** (Julien et al., 2013b), binding of PGT121 to core-xylosylated mono-antennary LacDiNAc *N*-glycan (glycan **28**) was studied using saturation-transfer difference-nuclear magnetic resonance (STD-NMR) in combination with computational methods (Schrödinger, 2016) (Friesner et al., 2004; Halgren et al., 2004). The intensities in the STD ^1H NMR spectrum correlate with the proximity of the ^1H nuclei of the glycan **28** to the surface of PGT121 in the bound state, hence providing structural information on the molecular recognition by depicting the proximity of ligand-protein contacts in the complex (the so-called STD binding epitope mapping of the ligand) (Angulo and Nieto, 2011; Mayer and Meyer, 2001). Here, the binding epitope map of glycan **28** as bound to PGT121 (Figures S2 and S3) showed that the terminal GalNAc and GlcNAc rings (residues 7 and 8 in Figure 2A) have the closest contacts to the protein surface, while the rest of the glycan shows a lower but homogeneous degree of proximity to the surface of PGT121, supporting that all the sugar residues of the glycan are involved in the interaction with PGT121. The binding epitope map obtained by STD-NMR allowed to experimentally validate a computational three-dimensional (3D) model obtained by docking calculations followed by molecular dynamics (MD) simulations. The lowest energy docking solution (Figure 2A) showed very good agreement with the experimental STD binding epitope mapping. A 100 ns MD simulation run (Figure 2B) starting from the docking model showed that the central region of glycan **28** lower mobility with all the sugar residues making contact to the PGT121 surface, whereas the reducing end shows a higher degree of flexibility, explaining the slightly lower STD values observed at the reducing GlcNAc. Structural analysis of PGT121 Fab binding to the self bi-antennary glycan **144SIA** has been previously reported (Julien et al., 2013b). Globally, the results strongly support that glycan **28** binds to PGT121 in a comparable manner to this self-glycan (Figure 2C) and that the non-self components of glycan **28** are not only tolerated in the binding site but form contacts with the antibody Fab region.

To support the NMR and modeling analysis, IgG variants of PGT121 containing alanine mutations at positions previously identified as important for glycan recognition were tested on the synthetic parasite glycan microarray (Figure 2E) (Julien et al., 2013b). Paratope mutations D31A, K53A, D56A, H97A, and R99A were previously shown to greatly reduce binding to self bi-antennary sialylated complex *N*-glycans (Julien et al., 2013b) (Figure 2D). However, these mutations did not universally abolish glycan binding to the synthetic parasite glycan structures. Although K53A, D56A, H97A, and R100A abolished binding to the majority of glycan structures, binding to mono- and bi-antennary glycans **28**, **59**, **63**, **126**, **128**, and **133** was retained with the R99A and D31A mutants. Interestingly, mutation of K53, which was acquired during somatic hypermutation (Garces et al., 2015), abolished binding to all but **128**, which has a non-self terminal LacDiNAc motif, suggesting that binding to the non-self sugar could have been present prior to self-glycan recognition.

Binding of HIV-1 glycan-reactive bnAbs to whole cercariae and soluble glycoproteins extracted from the different *S. mansoni* life stages

Having shown that glycan-reactive bnAbs bind to both mammalian self and non-self glycan motifs present on different *S. mansoni* life stages in the context of glycan microarrays, we next investigated whether these bnAbs also recognized these glycan structures in a more native form by performing western blot (WB) analysis and ELISA with solubilized glycoprotein antigens from different life stages. Because of the heterogeneous nature of the pathogen lysates, these assays were used to provide a qualitative assessment of cross-pathogen reactivity. Solubilized antigens from cercariae (SCA) and adult worms (AWA) (Figures 3A and S4A), as well as recombinant gp120 (Figure S4B), were separated using SDS-PAGE and transferred onto a cellulose membrane for staining. Sera from an *S. mansoni*-infected individual detected numerous bands in the SCA and AWA lysates (Figure S4A). Glycan-reactive mAbs 2G12, PGT121, PGT128, and PGT151 showed reactivity to both SCA and AWA with different patterns of antigen binding (Figure 3A). V3-specific HIV-1 mAb F425-b4e8, which does not bind glycans, showed no reactivity. BnAbs PGT121, PGT128, 2G12, PGT151 VRC01, and F425-b4e8 all showed binding to gp120 (Figure S4B).

Although denatured and reduced soluble antigens were used for the western blot, soluble antigens were kept in a native state for ELISA. N332/V3-specific bnAbs 2G12 and PGT121

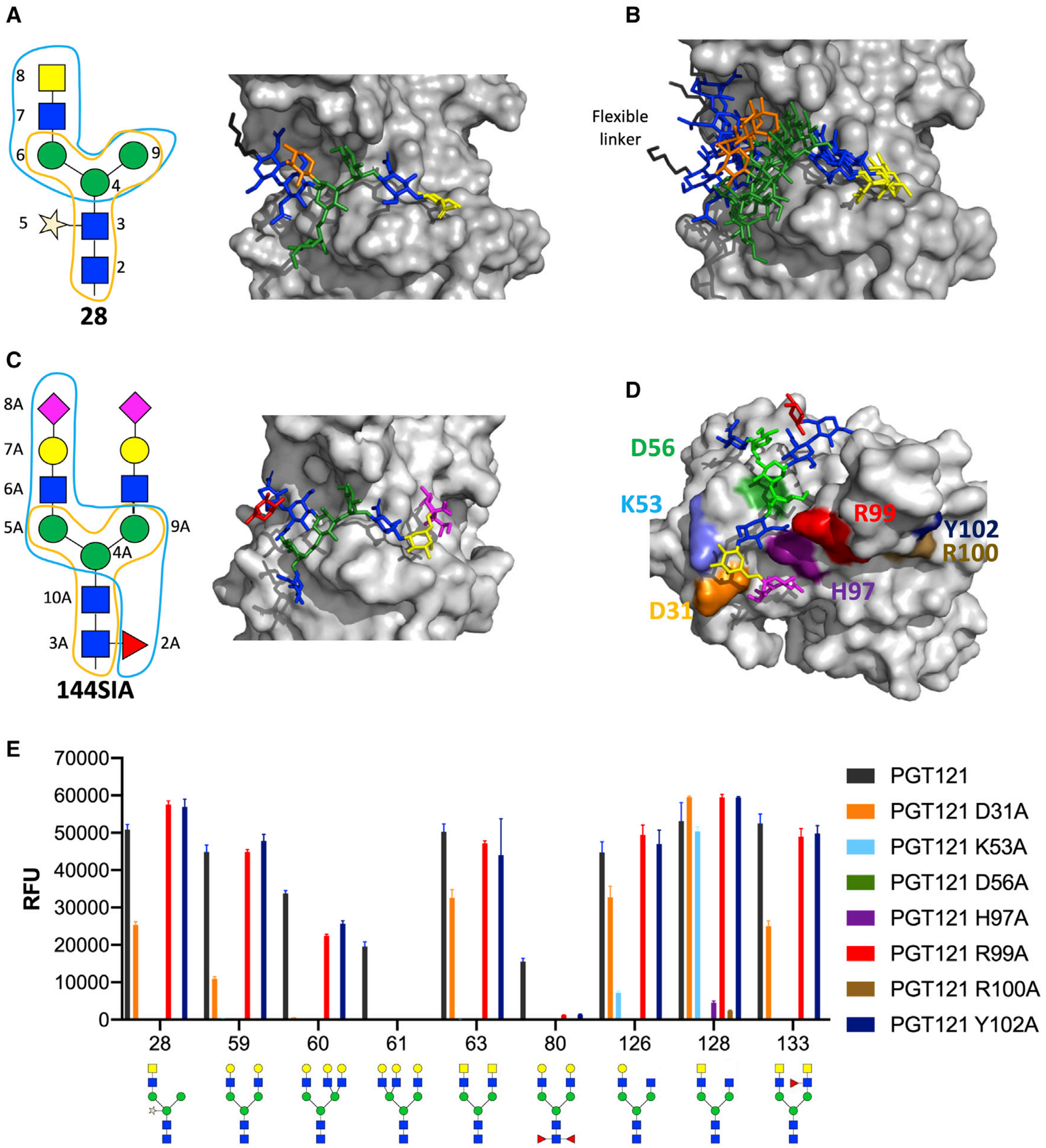

Figure 2. Binding mode of PGT121 to a xylosylated parasite *N*-glycan with terminal LacDiNAc and glycan-array binding of PGT121 alanine mutants

(A) Model after the solved STD-NMR structure of parasite *N*-glycan **28** with terminal LacDiNAc occupying the secondary binding pocket of PGT121, which is usually occupied by a bi-antennary complex-type *N*-glycan. Interactions were determined by STD-NMR (see Figures S2A–S2C and S3; Table S1). The conserved core of the *N*-glycan is indicated in orange. The part contacted by PGT121 is indicated in blue.

(B) Molecular dynamics and superposition of the parasite *N*-glycan **28** occupying the secondary binding pocket of PGT121. PDB files of representative frames of the MD simulation represented in (B) are found Data S1–S3.

(C) Structure of the complex *N*-glycan **144SIA**, bound by the secondary binding pocket of PGT121 (PDB: 4FQC) (Mouquet et al., 2012). The conserved core of the *N*-glycan is indicated in orange. The part contacted by PGT121 is indicated in blue.

(D) Structure of glycan **144SIA** bound in the secondary, open face of PGT121 (PDB: 4FQC) (Mouquet et al., 2012) with paratope residues color-coded according to alanine mutants used in (E).

(E) Alanine-knockout mutants covering the complex-glycan binding, open face of IgG PGT121 were screened on the synthetic glycan array for parasite glycan recognition. Binding is reported in relative fluorescence units (RFU). Each histogram represents the average RFU values for four replicates spots and the error bars represent the SD of the mean. Man, green circle; GlcNAc, blue square; GalNAc, yellow square; Gal, yellow circle; sialic acid, purple diamond; fucose, red triangle; xylose, star.

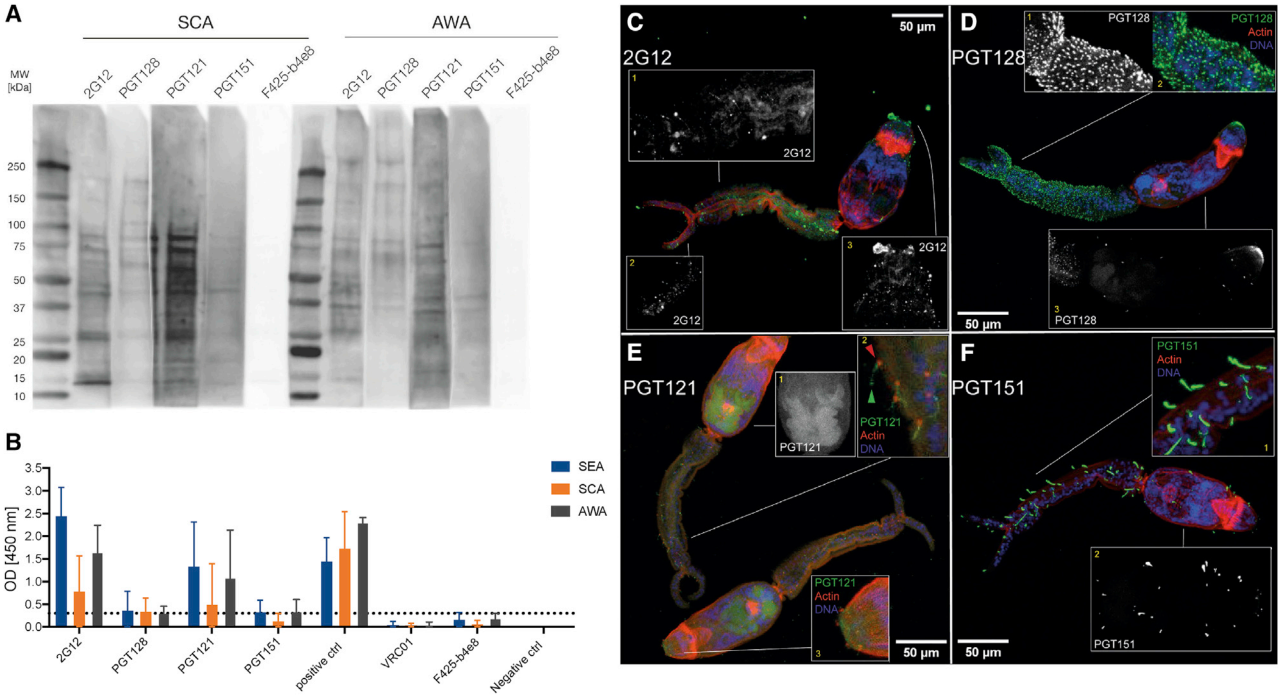

Figure 3. Cross-reactivity of HIV-1 bnAbs with *S. mansoni*

(A) Western blot of *S. mansoni*-soluble cercariae antigen (SCA) and adult worm antigen (AWA) (both at 25 µg per lane) with glycan-binding bnAbs 2G12, PGT128, PGT121, and PGT151 and V3-specific mAb F425-b4e8 (all at 50 µg/mL). A control western blot stained with sera from *S. mansoni*-infected humans or rabbits immunized with SCA/AWA is shown in Figure S4A. Binding of HIV-1 bnAbs to recombinant gp120 is shown in Figure S4B.

(B) Optical density at 450 nm of 2G12, PGT128, PGT121, and PGT151 (at 50 µg/mL) determined by ELISA using *S. mansoni*-soluble egg antigen (SEA), SCA, and AWA. V3-specific mAb F425-b4e8 and CD4-bnAb VRC01 (at 50 µg/mL), as well as healthy human serum, and *S. mansoni*-positive human serum as positive control. Error bars represent data from repeated experiments. The horizontal dotted line indicates the cut-off for the positive control provided with the assay kit (see STAR Methods). Bar graphs show the mean of the OD values from at least 3 repeat experiments, and the error bars represent the SD.

(C–F) Binding of HIV-1 bnAbs to *S. mansoni* cercariae. HIV-1 bnAbs binding was detected with Alexa Fluor 488 (green), actin was detected with rhodamine (red), and DNA was stained with DAPI (blue). Black and white images show projections of bnAb staining only, while colored images show overlays. (C) Binding of 2G12 to *S. mansoni* cercariae. 2G12 bound to putative ciliated sensory papillae (**1**), superficial neural projections (**2**), and the duct opening on the oral sucker and dotted fractions of glycoprotein on the oral sucker (**3**). (D) Binding of PGT128 to *S. mansoni* cercariae. PGT128 recognized spines, ciliated sensory papillae, and basement membrane/tegument/glycocalyx structures (**1**), similar to ConA (Figure S5A). PGT128 also recognized surface motifs (spines, ciliated sensory papillae, and basement membrane/tegument/glycocalyx structures) above the actin layer (**2**) and the duct opening on the oral sucker and dotted fractions of glycoprotein on the oral sucker (**3**). (E) Binding of PGT121 to *S. mansoni* cercariae. PGT121 recognizes the pre- and post-acetabular gland system, which is sits interior of the parasite (**1**) and surface motifs of ciliated sensory papillae and basement membrane/tegument/glycocalyx structures above the actin layer (**2**). The green arrow indicates ciliated sensory papillae reaching outside while being anchored to the outer surface by actin (red arrow). PGT121 also bound the duct opening on the oral sucker and dotted fractions of glycoprotein, co-locating with actin on the oral sucker (**3**). (F) Binding of PGT151 to *S. mansoni* cercariae. PGT151 recognized surface motifs of ciliated sensory papillae above the actin layer with high intensity (**1**) and ciliated tufts of the flame cells and on the protone phridial tubules (**2**). Binding of glycan-independent HIV-1 bnAbs F425-b4e8 and VRC01 is shown in Figures S6A and S6B. Confocal microscopy staining was performed twice on different cercariae preparations. Representative images are shown from one experiment.

recognized SCA, AWA, and soluble egg antigen (SEA) with mean optical densities (OD) above the cut-off (3-fold above background) (Figure 3B). In contrast, N332/V3-specific bnAb PGT128 and gp41/gp120 interface bnAb PGT151 showed very weak binding. The lower binding of PGT151 to soluble antigens compared with the shotgun glycan microarray is likely related to the difference in glycan density and/or presentation of glycan ligands between the microarray and on native antigens. Glycan presentation has been shown to be critical for recognition by some HIV-1 bnAbs (Walker et al., 2011; Wang et al., 2008). Control bnAbs, VRC01, and F425-b4e8, as well as control healthy human sera provided with the kit, did not show binding above the cut-off.

Next, to examine HIV-1 bnAb cross-reactivity in a more biological setting, whole cercariae were stained with glycan-reactive bnAbs (PGT121, PGT128, PGT151, and 2G12), VRC01, F425-b4e8, ConA (a lectin specific for high-mannose and some hybrid glycans), or PHA-L (a lectin specific for non-sialylated tri- and tetra-antennary complex glycans), and binding was visualized using confocal microscopy. ConA recognized surface glycans with high intensity (Figure S5A), whereas PHA-L recognized structures within the cercariae with a lower intensity (Figure S5B). Glycan-binding bnAbs showed different patterns of binding. The binding pattern of PGT128 to cercariae was similar to that of ConA (Figure 3D) with binding observed to the oral sucker as well other surface motifs on the spines and sensory

papillae, suggesting a high density of high-mannose glycans. For PGT121, which binds complex-type *N*-glycans (Julien et al., 2013b), a different binding pattern was observed (Figure 3E), with binding predominantly observed against surface motifs of ciliated sensory papillae and basement membrane/tegument/ glycocalyx structures, as well as the internal pre- and post-acetabular gland system. 2G12 bound to the ciliated sensory papillae and superficial neural projections, and duct opening on the oral sucker and dotted fractions of glycoprotein on the oral sucker (Figure 3C). PGT151 showed intense binding to the ciliated tufts of the flame cells and ciliated regions on the protone phridial tubules (Figure 3F). No specific binding was observed for control mAbs F425-b4e8 (Figure S6A) and VRC01 (Figure S6B). Overall, HIV-1 glycan-binding bnAbs cross-react with a range of *S. mansoni* glycoproteins in both soluble and native conformations.

S. mansoni seroreactivity in the IAVI Protocol C bnAb cohort

Having demonstrated cross-reactivity of glycan-reactive HIV-1 bnAbs with *S. mansoni*, we next used plasma from an HIV-1 infection cohort to investigate whether a previous or current *S. mansoni* infection might associate with development of glycan-reactive HIV-1 bnAbs and/or enhanced neutralization breadth and potency. The International AIDS Vaccine Initiative (IAVI) Protocol C longitudinal HIV-1 infection cohort was selected for this analysis, as many of the trial locations were in *S. mansoni* endemic areas, and the development of neutralization breadth and the epitopes targeted by the bnAbs have been extensively studied previously (Landais et al., 2016). Neutralization breadth was previously measured on a cross-clade panel of HIV-1 pseudoviruses (92TH021 [CRF0-AE], 94UG103 [clade A], 92BR020 [clade B], JRCSF [clade B], IAVIC22 [clade C, also named MGRM-C026] and 93IN905 [clade C]) and a neutralization score representative of breadth and potency calculated.

Fifty-nine donors were selected from individuals measuring weak (neutralization score < 0.5), medium (neutralization score < 1 and ≥ 0.5), or top (neutralization score ≥ 1) neutralization scores as defined by Landais et al. (Landais et al., 2016) and were representative of the full cohort (n = 439). To determine a previous or current *S. mansoni* infection, we used a commercial ELISA kit to measure presence of IgG specific to soluble egg antigen (SEA). The presence of SEA-reactive antibodies was measured at three time points following HIV-1 infection (median of 24, 96, and 216 weeks post-infection) (Table S2). Forty-one percent (24/59) had *S. mansoni* SEA-reactive IgG detectable in at least one time point (Figure S7A). At time point 3 (TP3), the *S. mansoni*-reactive donors (termed SH1) had a higher percentage of top and medium neutralizers (75%) compared with non-*S. mansoni*-reactive donors (termed SH0) (54%) (Figure 4A). When considering the overall peak neutralization score detected for each donor as defined by Landais et al. (Landais et al., 2016), although the percentage of top neutralizers was similar between the SH0 and SH1 groups (37% and 42%, respectively), the percentage of weak neutralizers was 3 times higher in the SH0 group compared with the SH1 group (37% and 13% respectively). Overall, these differences were not statistically significant for this small cohort. The neutralizing activity in SH1 and SH0 donors was targeted toward similar neutralizing epitopes and showed similar levels of single and multi-epitope targeted specificity (Figure 4B).

Comparison of the neutralization score, neutralization breadth and geometric mean serum dilution that inhibits infection by 50% (ID_{50}) between SH1 and SH0 donors did not reveal statistically significant differences at any of the time points studied here (Figures 4C, S7B, and S7C), and those with *S. mansoni* seroreactivity at TP1 did not develop bnAbs at a faster rate. However, higher viral loads at setpoint ($p = 0.038$; Figure 4C) and longer time to anti-retroviral therapy (ART) ($p = 0.0082$; Figure 4C) for SH1 donors in comparison with SH0 donors were observed. There was no difference in $CD4^+$ T cell count at viral setpoint between these donor groups (Figure 4C). This is consistent with previous observations showing pre-existing *S. mansoni* infections raise the viral load at HIV-1 seroconversion (Downs et al., 2017), while donors with *S. mansoni* infection at seroconversion have been reported to progress to AIDS slower than those without, supporting these observations (Colombe et al., 2018). Overall, we observed a higher proportion of *S. mansoni* seroreactive donors (SH1) with medium or top neutralization compared with SH0 donors.

PCDN76 N332/V3 bnAb lineage cross-reacts with *S. mansoni*

IAVI protocol C donor PC076 has previously been shown to generate an N332/V3 bnAb response (Macleod et al., 2016), and our analysis showed that this donor was also *S. mansoni* SEA seropositive (standard units > 30) at 47, 95, and 216 weeks after HIV-1 infection (Table S2). PC076 had a neutralization score of 1.2 (geometric mean titer [GMT] 88 and neutralized 4 of 6 viruses in the indicator panel) and was therefore within the Protocol C top neutralization category. We therefore selected this donor to examine further whether *S. mansoni* antibody reactivity might contribute to glycan-reactive HIV-1 bnAb development. The bnAb lineage responsible for the plasma neutralizing activity, PCDN76, has been described previously (Macleod et al., 2016) and the predicted UCA (UCA-S and UCA-P, referring to last amino acid in CDRH3) were unable to bind autologous or heterologous gp120s (Macleod et al., 2016). Antibody lineage members isolated at >16 months post-infection were shown to bind $Man_9GlcNAc_2$ (glycan **142M9**) but not glycan **142M8** ($Man_8GlcNAc_2$) (Macleod et al., 2016).

To determine whether development of the PCDN76 bnAb lineage might have been directed by pre-existing glycan-reactive Abs raised against *S. mansoni*, we measured the reactivity of PCDN76 bnAb lineage members against *S. mansoni*-soluble antigens in ELISA and whole cercariae using confocal microscopy. Although UCA-P and UCA-S did not bind to HIV-1 gp120 (Figure 5A), both UCAs bound soluble *S. mansoni* antigens (SCA, AWA, and SEA) on ELISA (Figure 5B), as well as whole cercariae (Figures 5C and 5D). More highly mutated lineage members showed reduced binding to soluble *S. mansoni* antigens but strong binding to gp120 (Figures 5A and 5B). Binding to whole cercariae was observed for some mutated lineage members. 22A (Figure 5F) (V_HJ_H-mutation 10%) showed a pattern of binding similar to the UCA and ConA (Figures 5C and 5D; compare

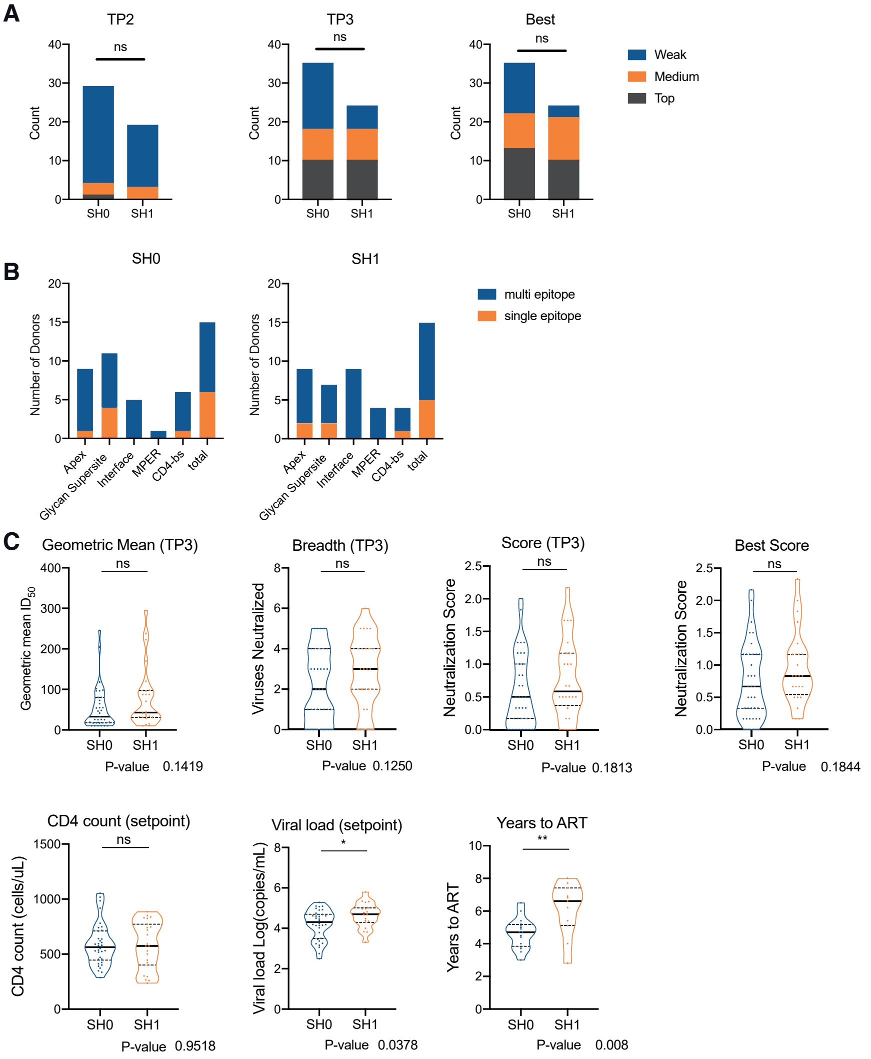

Figures 4. Analysis of HIV-1 neutralization and *S. mansoni* seroprevalence in IAVI Protocol C donors

(A) Comparison of neutralization score between donors with *S. mansoni* SEA seroreactivity (SH1) and no seroreactivity (SH0) at different time points after HIV-1 infection (TP2, TP3, and the time point with highest neutralization score [Best]). Neutralization score (Score) is a numerical representation of neutralization breadth and potency against a HIV-1 cross-clade virus panel and categorized by "TOP" (score $\geq$ 1), "MEDIUM" (score $<$ 1 and $\geq$0.5), and "WEAK" (score $<$ 0.5) as determined by Landais et al. (Landais et al., 2016). Groups were compared using a chi-square test, but no statistical differences were observed.

(B) Distribution of neutralizing epitopes targeted in sera (as determined by serum mapping studies; Landais et al., 2016) across *S. mansoni* status is displayed in a bar chart with the sum of each specificity overall. Single specificities are displayed in orange, while the distribution of mixed specificities is stacked on top in blue.

(C) Comparison of geometric mean ID_{50} at TP3, the number of viruses neutralized (breadth) at TP3, neutralization score at TP3, best neutralization score (from all time points in the study) (Landais et al., 2016), CD4 count at setpoint (cells per microliter), viral load (log RNA copies per milliliter) at setpoint, and time to ART in years from setpoint between donors with *S. mansoni* seroreactivity (SH1) and no *S. mansoni* seroreactivity (SH0). p values (Mann-Whitney test) are reported as follows: not significant (ns), *p $<$ 0.0332, **p $<$ 0.0021, ***p $<$ 0.0002, and ****p $<$ 0.00001. Additional cohort analysis can be found in Figures S7A–S7C.

Figure S4A), whereas 22C (V_HJ_H-mutation 18%) bound the spines and ciliated sensory papillae (Figure 5G). 16A (V_HJ_H-mutation 8%) (Figure 5E) and 38A (V_HJ_H-mutation 23%) (Figure 5H) displayed weak binding to whole cercariae, and no reactivity was observed for the most mutated bnAb 38B (V_HJ_H-mutation 26%) (Figure S6D). Therefore, despite the lack of gp120 binding, the UCA of the PCDN76 lineage shows reactivity toward *S. mansoni* antigens.

DISCUSSION

Elicitation of glycan-reactive HIV-1 bnAbs through vaccination is highly desirable, and although they are frequently found in sera/plasma of HIV-1-infected individuals displaying high neutralization breadth and potency, current state-of-the-art immunogens typically elicit Abs that bind the holes in the glycan shield rather than the glycans themselves. A key challenge in eliciting glycan-reactive bnAbs is the poor immunogenicity of the *N*-glycans on Env. In contrast, non-self glycans on other highly glycosylated pathogens can be strongly immunogenic.

Glycan-mediated cross-pathogen reactivity was previously described between HIV-1 bnAb 2G12 and *C. albicans* (Doores et al., 2010b; Dunlop et al., 2010; Luallen et al., 2008, 2009) and influenza (Lee et al., 2021). Although attempts were made to use yeast-derived immunogens to generate a glycan-reactive neutralizing antibody response, the lack of success was attributed to the specific requirement of antibody-domain exchange for 2G12 neutralizing activity (Doores et al., 2010b; Dunlop et al., 2010; Huber et al., 2010). Here we report the cross-reactivity of the more potent and broad second-generation glycan-reactive HIV-1 bnAbs with different life stages of *S. mansoni*. The observed cross-pathogen reactivity of HIV-1 bnAbs is facilitated by high-mannose and complex-type mammalian glycans as well as non-self glycan structures expressed by *S. mansoni*. For example, PGT128 binds high-mannose glycans on the shotgun parasite glycan array, to soluble *S. mansoni* antigens on ELISA and WB, and has a similar binding pattern with whole cercariae as the mannose-specific lectin ConA. In contrast, PGT121 binds to self- and non-self complex-type glycans on worms and to a lesser extent on cercariae and egg life stages of *S. mansoni*. STD-NMR and modeling of PGT121 binding to the non-self parasite *N*-glycan

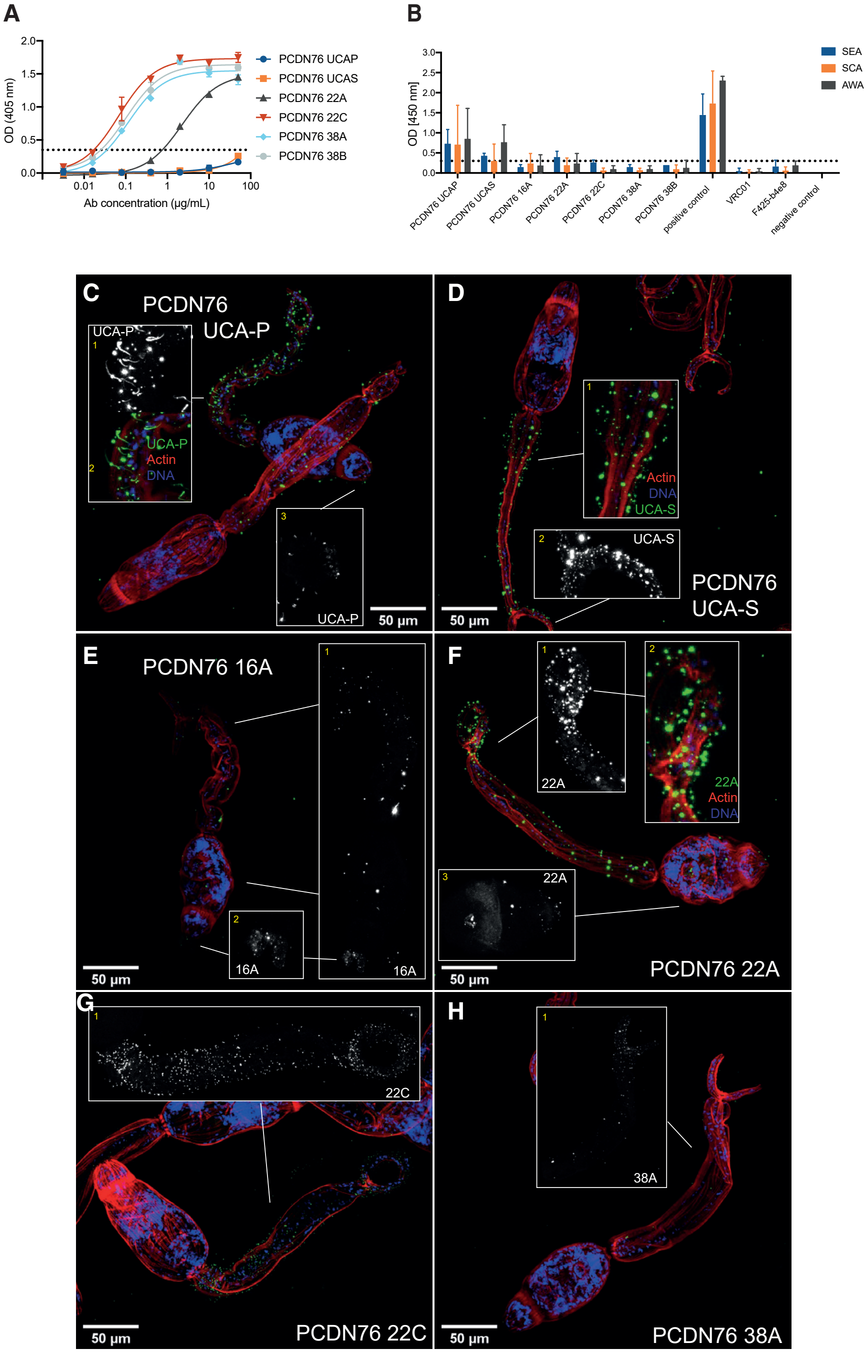

Figure 5. Cross-reactivity of N332/V3 PCDN76-lineage mAbs with *S. mansoni* isolates

(A) Binding of PCDN76 38A and 38B and their less mutated precursors UCA-P, UCA-S, 22A, and 22C to recombinant JR-CSF gp120 was measured using ELISA. Experiments were performed in duplicate and repeated twice. A representative dataset is shown. Error bars represent the range of the value for experiments performed in duplicate (not shown when smaller than symbol size).

(B) Binding of PCDN76 38A and 38B and their less mutated Ab precursors to *S. mansoni* SEA, SCA, and AWA on ELISA (at 50 μg/mL). *N*-glycan-independent HIV-1 mAbs F425-b4e8 and VRC01 were used as negative controls and *S. mansoni*-positive human serum as positive control. Error bars represent data from repeated experiments. Bar graphs show the mean of the OD values from at least 3 repeat experiments, and the error bars represent the SD.

(C–H) Binding of less mutated PCDN76 precursors (UCA-P, UCA-S, 22A, 22C, and 38A) to *S. mansoni* cercariae. HIV-1 mAbs were detected with Alexa Fluor 488 (green), actin was detected with rhodamine (red), and DNA was stained with DAPI (blue). Black and white images show projections of mAb staining only, while colored images show overlays. (C) UCA-P recognizes superficial neural projections and ciliated sensory papillae (**1**), similar to 2G12. UCA-P recognized motifs of superficial neural projections and ciliated sensory papillae are located above the actin layer (**2**). Similar to 2G12, UCA-P displays binding to duct opening on the oral sucker (**3**). (D) UCA-S recognizes surface motifs (spines, ciliated sensory papillae, and basement membrane/tegument/glycocalyx structures) above the actin layer (**1**), and basement membrane/tegument/glycocalyx structures similar to ConA and PGT128 (**2**). (E) 16A binds to glycoproteins on the body, which could be ciliated tufts of the flame cells and/or ciliated regions on the protone phridial tubules and to ciliated sensory papillae on the tail and the body (**1**). Furthermore, binding to the duct opening on the oral sucker is observed (**2**). (F) 22A recognizes spines, ciliated sensory papillae and basement membrane/tegument/glycocalyx structures, similar to ConA and PGT128 (**1**). 22A recognizes surface motifs (spines, ciliated sensory papillae and basement membrane/tegument/glycocalyx structures) above the actin layer (**2**). 22A binds dotted fractions of glycoprotein on the oral sucker (**3**). (G) 22C recognizes spines, ciliated sensory papillae, and basement membrane/tegument/glycocalyx structures (**1**). (H) 38A recognizes spines, ciliated sensory papillae, and basement membrane/tegument/glycocalyx structures. Confocal images showing staining of PGT128 and PCDN76 38B are shown in Figures S6C and S6D, respectively. Confocal microscopy staining was performed twice on different cercariae preparations. Representative images are shown from one experiment.

28 revealed that the non-self LacDiNAc and xylose motifs were not only tolerated but contributed to the antibody-glycan interaction in the secondary, open face binding site of PGT121.

Previous studies indicate that the predicted UCA of an HIV-1 bnAb lineage does not always interact with HIV-1 Env (Liao et al., 2013; Macleod et al., 2016), and therefore which antigen initially activated the UCA remains unclear in some cases. Although the predicated UCA of PCDN76 was previously shown to not bind autologous gp120 present at 5 months post-infection (Macleod et al., 2016), we demonstrated binding to whole

cercariae by confocal microscopy and soluble SCA, SEA, and AWA in ELISA. Furthermore, as the bnAb lineage undergoes affinity maturation against HIV-1 Env through somatic hypermutation, the cross-reactivity toward *S. mansoni* decreases, while the reactivity toward HIV-1 increases. These data could suggest that the PCDN76 germline was originally selected against another glycosylated pathogen, such as *S. mansoni* and that, upon infection with HIV-1, was re-activated because of cross-reactivity between glycan epitopes on HIV-1 Env. This lineage could then have undergone additional affinity maturation against HIV-1 Env to develop HIV-1-specific neutralization breadth and potency facilitated by viral escape and antibody somatic hypermutation. Additional experiments are needed to investigate this hypothesis further for donor PC076. A similar pattern on glycan-reactive bnAb evolution in SHIV-infected macaques has been reported by Haynes and co-workers, where the precursor antibodies of two glycan-reactive bnAb lineages (DH851 and DH898) bound to *C. albicans* and had very weak reactivity to HIV-1 Env (Williams et al., 2021). Class-switched and mutated clonally related VH genes were identified prior to SHIV infection, suggesting that other glycosylated antigens could have initiated these bnAbs lineages. They also identified a glycan-reactive antibody lineage (DH717) from Man_9-V3 glycopeptide-immunized macaques. Clonally related antibodies isolated prior to vaccination bind to *C. albicans* but lack reactivity with the Man_9-V3 immunogen, further supporting this hypothesis. Structural studies revealed that the DH851 and DH898 antibodies defined a new category of glycan-reactive dimerized antibodies where the mechanisms of Fab dimerization included disulfide bonds, hydrophobic interactions and hydrogen bonding rather than domain exchange observed for HIV-1 bnAb 2G12. Similar Fab-dimerized glycan-reactive (FDG) antibodies were also identified in SHIV-infected macaques, as well as HIV-1-naive individuals and SHIV-naive macaques. Structural studies of the PCDN76 lineage are required to determine the mechanism of PCDN76 UCA glycan recognition and whether Fab-dimers are formed that enhance the affinity of the glycan-antibody interaction.

A further example of cross-pathogen priming in the context of glycan antigens is suggested from sequence analysis of glycan-binding human mAbs isolated against *Klebsiella pneumoniae* that also efficiently neutralize *Pseudomonas luteola* lipopolysaccharide *in vitro* and bind HIV-1 Env (Rollenske et al., 2018). Unlike germline-encoded "natural Abs" that recognize glycan epitopes through low affinity, the cross-reactive *K. pneumoniae* mAbs had a high degree of somatic hypermutation (median 24 bp per IGVH gene), suggesting that the B cells have undergone repeated rounds of reactivation. Whether these reactivation events were achieved through re-infection with *K. pneumoniae* or from infection with another pathogen displaying similar glycan motifs is not known but could be suggestive of cross-pathogen priming.

Cross-antigen priming in the context of protein antigens or environmental antigens has also been reported for gp41-specific antibody development in HIV-1 infection (Liao et al., 2011; Trama et al., 2014) and vaccination (Williams et al., 2015). The initial antibodies elicited during the acute phase of an HIV-1 infection were shown to target gp41 and despite a relatively high level of SHM, were non-neutralizing. The UCA of these antibodies did not bind the early transmitted/founder virus but were polyreactive and bound non-HIV-1 protein antigens on gut microflora or host antigens, suggesting that the early gp41-specific antibody response arises from a pre-existing pool of mutated B cells. Haynes and co-workers studied these gp41 antibodies in the context of Env vaccination (Env DNA prime and Env rAd5 boost) and showed that gp41-specific non-neutralizing antibodies, derived from gp41-intestinal microbiota cross-reactive B cells, dominated the vaccine (Williams et al., 2015) response. Unlike the studies outlined above for glycan antigens, the pre-existing gp41 reactive B cells divert the antibody response generated by Env vaccination to non-neutralizing epitopes on HIV-1 Env.

Although a higher frequency of HIV-1-infected individuals with *S. mansoni* seropositivity developed an HIV-1 neutralizing antibody response, the data presented does not suggest that this would be a dominant mechanism in which HIV-1 glycan-binding bnAb arise, as *S. mansoni*-seropositive donors generated bnAbs targeting a range of Env epitopes. However, the observations reported here could be applicable in HIV-1 vaccine development aimed at eliciting glycan-binding bnAbs. Taking advantage of the molecular mimicry observed between HIV-1 Env *N*-glycans and *S. mansoni N*-glycans, *S. mansoni* glycoprotein antigens or immunogens displaying non-self parasite glycans could be used to prime a glycan-specific antibody response that is subsequently boosted and shepherded to become HIV-1 broadly neutralizing with Env-based immunogens. Therefore, an important next step will be to determine whether immunogens displaying parasite glycans (either glycoproteins derived from *S. mansoni* or chemically modified HIV-1 Env-based immunogens) are able elicit an antibody response that cross-reacts with HIV-1 Env glycans.

Limitations of the study

This study focuses on cross-reactivity of HIV-1 bnAbs with *S. mansoni*, which is not globally endemic. Therefore, further research is needed to study cross-reactivity of HIV-1 glycan-binding bnAbs with more widely prevalent highly glycosylated pathogens, which could also play a role in the development of glycan-binding HIV-1 bnAbs. A further limitation of this study is that binding to multimeric forms of autologous trimers by the UCA of PCDN76, which may have higher affinity for precursor B cells, have not been tested.

CONSORTIA

The IAVI Protocol C Investigators Susan Allen (Emory University, USA and ZERHP, Zambia), William Kilembe (ZEHRP, Zambia), Shabir Lakhi (ZEHRP, Zambia), Mubiana Inambao (ZEHRP, Zambia), Etienne Karita (Rwanda-Zambia HIV Research Group, Project San Francisco, Kigali, Rwanda), Anatoli Kamali (MRC/UVRI Uganda), Eduard J. Sanders (KEMRI, Kenya and Oxford University, UK), Omu Anzala (KAVI, Kenya), Vinodh Edward (The Aurum Institute, South Africa), Linda-Gail Bekker (Cape Town University, South Africa), Jianming Tang (University of Alabama Birmingham, USA).

The IAVI African HIV Research Network also includes Jill Gilmour (IAVI and London Imperial College, UK), Eric Hunter (Emory University, USA), Matt Price (IAVI and University of California San Francisco, USA).

STAR★METHODS

Detailed methods are provided in the online version of this paper and include the following:

- KEY RESOURCES TABLE
- RESOURCE AVAILABILITY
  - Lead contact
  - Materials availability
  - Data and code availability
- EXPERIMENTAL MODEL AND SUBJECT DETAILS
  - Human subjects
  - Ethics for protocol C
- METHOD DETAILS
  - bnAbs incubation on a synthetic glycan microarray
  - bnAbs incubation on a shotgun parasite glycan microarray
  - NMR spectroscopy
  - Molecular docking and molecular dynamics simulations
  - Measurement of SCA, AWA and gp120 binding by Western blot
  - ELISA for SEA
  - ELISA for SCA and AWA
  - *S. mansoni* cercariae staining
  - Gp120 ELISA
- QUANTIFICATION AND STATISTICAL ANALYSIS

SUPPLEMENTAL INFORMATION

Supplemental information can be found online at https://doi.org/10.1016/j.celrep.2022.110611.

ACKNOWLEDGMENTS

This project has received funding from the European Union's Horizon 2020 Research and Innovation program under grant agreement 681137 (to K.J.D. and I.H.), the Medical Research Council (MRC) (to K.J.D. [MR/K024426/1]), The Rosetrees Trust (to K.J.D. [M686]) and Fondation Dormeur, Vaduz (to K.J.D). This research was funded or supported by the National Institute for Health Research Biomedical Research Centre based at Guy's and St Thomas' NHS Foundation Trust and King's College London and/or the NIHR Clinical Research Facility. The views expressed are those of the authors and not necessarily those of the National Health Service (NHS), the National Institute for Health Research (NIHR), or the Department of Health. N.R. acknowledges funding from Ministry of Science and Education grants CTQ2017-90039-R, RTC-2017-6126-1, and CTQ2011-27874 (fellowship to K.B.) and the Maria de Maeztu Units of Excellence Program from the Spanish State Research Agency (grant MDM-2017-0720). F.A. was funded by the Wellcome Trust (104958/Z/14/Z). J.A. was supported by the Spanish Ministry of Science, Innovation and Universities through the grant PID2019-109395GB-I00. J.A. and S.M. acknowledge support of BBSRC (grant BB/P010660/1). T.H. and S.W. were funded by Biotechnology and Biological Sciences Research Council (BBSRC) Norwich Research Park Doctoral Training Grant BB/M011216/1. IAVI's work is made possible by generous support from many donors, including the Bill & Melinda Gates Foundation, the Ministry of Foreign Affairs of Denmark, Irish Aid, the Ministry of Finance of Japan in partnership with The World Bank, the Ministry of Foreign Affairs of the Netherlands, the Norwegian Agency for Development Cooperation, the United Kingdom Department for International Development (DFID), and the United States Agency for International Development. The full list of IAVI donors is available at www.iavi.org.

Brendan McAtarsney and Jonathan Hare from the IAVI Human Immunology Lab (HIL) for coordinating the samples transfers and shipments.

Monica Agromayor and the KCL Nikon Centre for assistance and advice on confocal microscopy. NMRI strain *Schistosoma mansoni*-infected *Biomphalaria glabrata* snails were provided by the NIAID Schistosomiasis Resource Center, Rockville, USA.

AUTHOR CONTRIBUTIONS

Conceptualization, K.J.D., S.A.K., I.H., and N.R.; Methodology, I.H., S.A.K., S.S., K.B., S.M., S.W., A.v.D., S.K., T.H., F.A., and E.L.; Formal Analysis, K.J.D., I.H., S.S., S.M., S.W., A.v.D., and E.L.; Resources, F.A., A.M.E., E.L., IAVI Protocol C Investigators; Supervision, K.J.D., A.M.E., C.H.H., J.A., and N.R.; Writing – Original Draft, K.J.D. and I.H.; Writing – Review & Editing, all authors.

DECLARATION OF INTERESTS

The authors declare no competing interests.

Received: October 21, 2021
Revised: January 26, 2022
Accepted: March 11, 2022
Published: April 5, 2022

REFERENCES

Amornkul, P.N., Karita, E., Kamali, A., Rida, W.N., Sanders, E.J., Lakhi, S., Price, M.A., Kilembe, W., Cormier, E., Anzala, O., et al. (2013). Disease progression by infecting HIV-1 subtype in a seroconverter cohort in sub-Saharan Africa. AIDS *27*, 2775–2786.

Angulo, J., and Nieto, P.M. (2011). STD-NMR: application to transient interactions between biomolecules-a quantitative approach. Eur. Biophys. J. *40*, 1357–1369.

Astronomo, R.D., and Burton, D.R. (2010). Carbohydrate vaccines: developing sweet solutions to sticky situations? Nat. Rev. Drug Discov. *9*, 308–324.

Behrens, A.J., Vasiljevic, S., Pritchard, L.K., Harvey, D.J., Andev, R.S., Krumm, S.A., Struwe, W.B., Cupo, A., Kumar, A., Zitzmann, N., et al. (2016). Composition and antigenic effects of individual glycan sites of a trimeric HIV-1 envelope glycoprotein. Cell Rep. *14*, 2695–2706.

De Boer, A.R., Hokke, C.H., Deelder, A.M., and Wuhrer, M. (2007). General microarray technique for immobilization and screening of natural glycans. Anal. Chem. *79*, 8107–8113.

Bonsignori, M., Kreider, E.F., Fera, D., Meyerhoff, R.R., Bradley, T., Wiehe, K., Alam, S.M., Aussedat, B., Walkowicz, W.E., Hwang, K., et al. (2017). Staged induction of HIV-1 glycan–dependent broadly neutralizing antibodies. Sci. Transl. Med. *9*, 1–26.

Brzezicka, K., Echeverria, B.A., Serna, S., Van Diepen, A., Hokke, C.H., and Reichardt, N.-C. (2015). Synthesis and microarray-assisted binding studies of core xylose and fucose containing N-glycans. ACS Chem. Biol. *10*, 1290–1302.

Burton, D.R., Walker, L.M., Phogat, S.K., Chan-Hui, P.Y., Wagner, D., Phung, P., Goss, J.L., Wrin, T., Simek, M.D., Fling, S., et al. (2009). Broad and potent neutralizing antibodies from an african donor reveal a new HIV-1 vaccine target. Science *326*, 285–289.

Cavacini, L., Duval, M., Song, L., Sangster, R., Xiang, S., Sodroski, J., and Posner, M. (2003). Conformational changes in env oligomer induced by an antibody dependent on the V3 loop base. AIDS *17*, 685–689.

Clark, B.E., Auyeung, K., Fregolino, E., Parrilli, M., Lanzetta, R., De Castro, C., and Pantophlet, R. (2012). A bacterial lipooligosaccharide that naturally

mimics the epitope of the HIV-neutralizing antibody 2G12 as a template for vaccine design. Chem. Biol. *19*, 254–263.

Collins, J.J., King, R.S., Cogswell, A., Williams, D.L., and Newmark, P.A. (2011). An atlas for Schistosoma mansoni organs and life-cycle stages using cell type-specific markers and confocal microscopy. PLoS Negl. Trop. Dis. *5*, e1009.

Colombe, S., Machemba, R., Mtenga, B., Lutonja, P., Kalluvya, S.E., de Dood, C.J., Hoekstra, P.T., van Dam, G.J., Corstjens, P.L.A.M., Urassa, M., et al. (2018). Impact of schistosome infection on long-term HIV/AIDS outcomes. PLoS Negl. Trop. Dis. *12*, e0006613.

van Diepen, A., Smit, C.H., van Egmond, L., Kabatereine, N.B., Pinot de Moira, A., Dunne, D.W., and Hokke, C.H. (2012). Differential anti-glycan antibody responses in Schistosoma mansoni-infected children and adults studied by shotgun glycan microarray. PLoS Negl. Trop. Dis. *6*, e1922.

van Diepen, A., van der Plas, A.J., Kozak, R.P., Royle, L., Dunne, D.W., and Hokke, C.H. (2015). Development of a Schistosoma mansoni shotgun O-glycan microarray and application to the discovery of new antigenic schistosome glycan motifs. Int. J. Parasitol. *45*, 465–475.

Doores, K.J., Bonomelli, C., Harvey, D.J., Vasiljevic, S., Dwek, R.A., Burton, D.R., Crispin, M., and Scanlan, C.N. (2010a). Envelope glycans of immunodeficiency virions are almost entirely oligomannose antigens. Proc. Natl. Acad. Sci. U S A *107*, 13800–13805.

Doores, K.J., Fulton, Z., Huber, M., Wilson, I.A., and Burton, D.R. (2010b). Antibody 2G12 recognizes di-mannose equivalently in domain- and nondomain-exchanged forms but only binds the HIV-1 glycan shield if domain exchanged. J. Virol. *84*, 10690–10699.

Doores, K.J., Kong, L., Krumm, S.A., Le, K.M., Sok, D., Laserson, U., Garces, F., Poignard, P., Wilson, I.A., and Burton, D.R. (2015). Two classes of broadly neutralizing antibodies within a single lineage directed to the high-mannose patch of HIV envelope. J. Virol. *89*, 1105–1118.

Doria-Rose, N.A., Schramm, C.A., Gorman, J., Moore, P.L., Bhiman, J.N., DeKosky, B.J., Ernandes, M.J., Georgiev, I.S., Kim, H.J., Pancera, M., et al. (2014). Developmental pathway for potent V1V2-directed HIV-neutralizing antibodies. Nature *508*, 55–62.

Downs, J.A., Dupnik, K.M., van Dam, G.J., Urassa, M., Lutonja, P., Kornelis, D., de Dood, C.J., Hoekstra, P., Kanjala, C., Isingo, R., et al. (2017). Effects of schistosomiasis on susceptibility to HIV-1 infection and HIV-1 viral load at HIV-1 seroconversion: a nested case-control study. PLoS Negl. Trop. Dis. *11*, e0005968.

Dubrovskaya, V., Tran, K., Ozorowski, G., Guenaga, J., Wilson, R., Bale, S., Cottrell, C.A., Turner, H.L., Seabright, G., O'Dell, S., et al. (2019). Vaccination with glycan-modified HIV NFL envelope trimer-liposomes elicits broadly neutralizing antibodies to multiple sites of vulnerability. Immunity *51*, 915–929.e7.

Dunlop, D.C., Ulrich, A., Appelmelk, B.J., Burton, D.R., Dwek, R.A., Zitzmann, N., and Scanlan, C.N. (2008). Antigenic mimicry of the HIV envelope by AIDS-associated pathogens. AIDS *22*, 2214–2217.

Dunlop, D.C., Bonomelli, C., Mansab, F., Vasiljevic, S., Doores, K.J., Wormald, M.R., Palma, A.S., Feizi, T., Harvey, D.J., Dwek, R.A., et al. (2010). Polysaccharide mimicry of the epitope of the broadly neutralizing anti-HIV antibody, 2G12, induces enhanced antibody responses to self oligomannose glycans. Glycobiology *20*, 812–823.

Echeverria, B., Serna, S., Achilli, S., Vivès, C., Pham, J., Thépaut, M., Hokke, C.H., Fieschi, F., and Reichardt, N.C. (2018). Chemoenzymatic synthesis of N-glycan positional isomers and evidence for branch selective binding by monoclonal antibodies and human C-type lectin receptors. ACS Chem. Biol. *13*, 2269–2279.

Falkowska, E., Ramos, A., Feng, Y., Zhou, T., Moquin, S., Walker, L.M., Wu, X., Seaman, M.S., Wrin, T., Kwong, P.D., et al. (2012). PGV04, an HIV-1 gp120 CD4 binding site antibody, is broad and potent in neutralization but does not induce conformational changes characteristic of CD4. J. Virol. *86*, 4394–4403.

Falkowska, E., Le, K.M., Ramos, A., Doores, K.J., Lee, J.H., Blattner, C., Ramirez, A., Derking, R., vanGils, M.J., Liang, C.H., et al. (2014). Broadly neutralizing HIV antibodies define a glycan-dependent epitope on the prefusion conformation of gp41 on cleaved envelope trimers. Immunity *40*, 657–668.

Freund, N.T., Wang, H., Scharf, L., Nogueira, L., Horwitz, J.A., Bar-On, Y., Golijanin, J., Sievers, S.A., Sok, D., Cai, H., et al. (2017). Coexistence of potent HIV-1 broadly neutralizing antibodies and antibody-sensitive viruses in a viremic controller. Sci. Transl. Med. *9*, 139–148.

Friesner, R.A., Banks, J.L., Murphy, R.B., Halgren, T.A., Klicic, J.J., Mainz, D.T., Repasky, M.P., Knoll, E.H., Shelley, M., Perry, J.K., et al. (2004). Glide: a new approach for rapid, accurate docking and scoring. 1. method and assessment of docking accuracy. J. Med. Chem. *47*, 1739–1749.

Garces, F., Lee, J.H., de Val, N., Torrents de la Pena, A., Kong, L., Puchades, C., Hua, Y., Stanfield, R.L., Burton, D.R., Moore, J.P., et al. (2015). Affinity maturation of a potent family of HIV antibodies is primarily focused on accommodating or avoiding glycans. Immunity *43*, 1053–1063.

Gray, E.S., Madiga, M.C., Hermanus, T., Moore, P.L., Wibmer, C.K., Tumba, N.L., Werner, L., Mlisana, K., Sibeko, S., Williamson, C., et al. (2011). The neutralization breadth of HIV-1 develops incrementally over four years and is associated with CD4+ T cell decline and high viral load during acute infection. J. Virol. *85*, 4828–4840.

Halgren, T.A., Murphy, R.B., Friesner, R.A., Beard, H.S., Frye, L.L., Pollard, W.T., and Banks, J.L. (2004). Glide: a new approach for rapid, accurate docking and scoring. 2. Enrichment factors in database screening. J. Med. Chem. *47*, 1750–1759.

Hraber, P., Seaman, M.S., Bailer, R.T., Mascola, J.R., Montefiori, D.C., and Korber, B.T. (2014). Prevalence of broadly neutralizing antibody responses during chronic HIV-1 infection. AIDS *28*, 163–169.

Huang, J., Ofek, G., Laub, L., Louder, M.K., Doria-Rose, N.A., Longo, N.S., Imamichi, H., Bailer, R.T., Chakrabarti, B., Sharma, S.K., et al. (2012). Broad and potent neutralization of HIV-1 by a gp41-specific human antibody. Nature *491*, 406–412.

Huber, M., Le, K.M., Doores, K.J., Fulton, Z., Stanfield, R.L., Wilson, I.A., and Burton, D.R. (2010). Very few substitutions in a germ line antibody are required to initiate significant domain exchange. J. Virol. *84*, 10700–10707.

Jones, C. (2005). Vaccines based on the cell surface carbohydrates of pathogenic bacteria. Acad. Bras. Cienc. *77*, 293–324.

Julien, J.-P., Cupo, A., Sok, D., Stanfield, R.L., Lyumkis, D., Deller, M.C., Klasse, P.-J., Burton, D.R., Sanders, R.W., Moore, J.P., et al. (2013a). Crystal structure of a soluble cleaved HIV-1 envelope trimer. Science *342*, 1477–1483.

Julien, J.P., Sok, D., Khayat, R., Lee, J.H., Doores, K.J., Walker, L.M., Ramos, A., Diwanji, D.C., Pejchal, R., Cupo, A., et al. (2013b). Broadly neutralizing antibody PGT121 allosterically modulates CD4 binding via recognition of the HIV-1 gp120 V3 base and multiple surrounding glycans. PLoS Pathog. *9*, e1003342.

Landais, E., Huang, X., Havenar-Daughton, C., Murrell, B., Price, M.A., Wickramasinghe, L., Ramos, A., Bian, C.B., Simek, M., Allen, S., et al. (2016). Broadly neutralizing antibody responses in a large longitudinal sub-saharan HIV primary infection cohort. PLoS Pathog. *12*, e1005369.

Lee, C.-C.D., Watanabe, Y., Wu, N.C., Han, J., Kumar, S., Pholcharee, T., Seabright, G.E., Allen, J.D., Lin, C.-W., Yang, J.-R., et al. (2021). A cross-neutralizing antibody between HIV-1 and influenza virus. PLoS Pathog. *17*, e1009407.

Liao, H.X., Chen, X., Munshaw, S., Zhang, R., Marshall, D.J., Vandergrift, N., Whitesides, J.F., Lu, X., Yu, J.S., Hwang, K.K., et al. (2011). Initial antibodies binding to HIV-1 gp41 in acutely infected subjects are polyreactive and highly mutated. J. Exp. Med. *208*, 2237–2249.

Liao, H.X., Lynch, R., Zhou, T., Gao, F., Munir Alam, S., Boyd, S.D., Fire, A.Z., Roskin, K.M., Schramm, C.A., Zhang, Z., et al. (2013). Co-evolution of a broadly neutralizing HIV-1 antibody and founder virus. Nature *496*, 469–476.

Luallen, R.J., Lin, J., Fu, H., Cai, K.K., Agrawal, C., Mboudjeka, I., Lee, F.-H., Montefiori, D., Smith, D.F., Doms, R.W., et al. (2008). An engineered Saccharomyces cerevisiae strain binds the broadly neutralizing human immunodeficiency virus type 1 antibody 2G12 and elicits mannose-specific gp120-binding antibodies. J. Virol. *82*, 6447–6457.

Luallen, R.J., Fu, H., Agrawal-Gamse, C., Mboudjeka, I., Huang, W., Lee, F.-H., Wang, L.-X., Doms, R.W., and Geng, Y. (2009). A yeast glycoprotein shows

CellPress
OPEN ACCESS

high-affinity binding to the broadly neutralizing human immunodeficiency virus antibody 2G12 and inhibits gp120 interactions with 2G12 and DC-SIGN. J. Virol. *83*, 4861–4870.

MacCalman, T.E., Phillips-Jones, M.K., and Harding, S.E. (2020). Glycoconjugate vaccines: some observations on carrier and production methods. Biotechnol. Genet. Eng. Rev., 1–33.

Macleod, D.T., Choi, N.M., Briney, B., Garces, F., Ver, L.S., Landais, E., Murrell, B., Wrin, T., Kilembe, W., Liang, C.-H., et al. (2016). Early antibody lineage diversification and independent limb maturation lead to broad HIV-1 neutralization targeting the env high-mannose patch. Immunity *44*, 1215–1226.

Maier, J.A., Martinez, C., Kasavajhala, K., Wickstrom, L., Hauser, K.E., and Simmerling, C. (2015). ff14SB: improving the accuracy of protein side chain and backbone parameters from ff99SB. J. Chem. Theor. Comput. *11*, 3696–3713.

Mascola, J.R., Lewis, M.G., Stiegler, G., Harris, D., VanCott, T.C., Hayes, D., Louder, M.K., Brown, C.R., Sapan, C.V., Frankel, S.S., et al. (1999). Protection of macaques against pathogenic simian/human immunodeficiency virus 89.6PD by passive transfer of neutralizing antibodies. J. Virol. *73*, 4009–4018.

Mayer, M., and Meyer, B. (2001). Group epitope mapping by saturation transfer difference NMR to identify segments of a ligand in direct contact with a protein receptor. J. Am. Chem. Soc. *123*, 6108–6117.

Mccoy, L.E., Van Gils, M.J., Ozorowski, G., Ward, A.B., Sanders, R.W., and Burton, D.R. (2016). Holes in the glycan shield of the native HIV envelope are a target of trimer-elicited neutralizing antibodies. Cell Rep. *16*, 1–12.

Mettu, R., Chen, C.Y., and Wu, C.Y. (2020). Synthetic carbohydrate-based vaccines: challenges and opportunities. J. Biomed. Sci. *27*, 9.

Moldt, B., Rakasz, E.G., Schultz, N., Chan-Hui, P.Y., Swiderek, K., Weisgrau, K.L., Piaskowski, S.M., Bergman, Z., Watkins, D.I., Poignard, P., et al. (2012). Highly potent HIV-specific antibody neutralization in vitro translates into effective protection against mucosal SHIV challenge in vivo. Proc. Natl. Acad. Sci. U S A *109*, 18921–18925.

Mouquet, H., Scharf, L., Euler, Z., Liu, Y., Eden, C., Scheid, J.F., Halper-Stromberg, A., Gnanapragasam, P.N.P., Spencer, D.I.R., Seaman, M.S., et al. (2012). Complex-type N-glycan recognition by potent broadly neutralizing HIV antibodies. Proc. Natl. Acad. Sci. U S A *109*, E3268–E3277.

Pauthner, M.G., Nkolola, J.P., Havenar-Daughton, C., Murrell, B., Reiss, S.M., Bastidas, R., Prévost, J., Nedellec, R., von Bredow, B., Abbink, P., et al. (2019). Vaccine-induced protection from homologous tier 2 SHIV challenge in nonhuman primates depends on serum-neutralizing antibody titers. Immunity *50*, 241–252.e6.

Pritchard, L.K., Harvey, D.J., Bonomelli, C., Crispin, M., and Doores, K.J. (2015). Cell- and protein-directed glycosylation of native cleaved HIV-1 envelope. J. Virol. *89*, 8932–8944.

Ringe, R.P., Pugach, P., Cottrell, C.A., LaBranche, C.C., Seabright, G.E., Ketas, T.J., Ozorowski, G., Kumar, S., Schorcht, A., van Gils, M.J., et al. (2018). Closing and opening holes in the glycan shield of HIV-1 envelope glycoprotein SOSIP trimers can redirect the neutralizing antibody response to the newly unmasked epitopes. J. Virol. *93*, e01656-18.

Rollenske, T., Szijarto, V., Lukasiewicz, J., Guachalla, L.M., Stojkovic, K., Hartl, K., Stulik, L., Kocher, S., Lasitschka, F., Al-Saeedi, M., et al. (2018). Cross-specificity of protective human antibodies against Klebsiella pneumoniae LPS O-antigen. Nat. Immunol. *19*, 617–624.

Sanders, R.W., Van Gils, M.J., Derking, R., Sok, D., Ketas, T.J., Burger, J.A., Ozorowski, G., Cupo, A., Simonich, C., Goo, L., et al. (2015). HIV-1 neutralizing antibodies induced by native-like envelope trimers. Science *244*, aac4223.

Schrödinger LLC (2016). Schrödinger Release 2016-4: MacroModel, Schrödinger, LLC, New York, NY, 2016.

Simek, M.D., Rida, W., Priddy, F.H., Pung, P., Carrow, E., Laufer, D.S., Lehrman, J.K., Boaz, M., Tarragona-Fiol, T., Miiro, G., et al. (2009). Human immunodeficiency virus type 1 elite neutralizers: individuals with broad and potent neutralizing activity identified by using a high-throughput neutralization assay together with an analytical selection algorithm. J. Virol. *83*, 7337–7348.

Sok, D., Pauthner, M., Briney, B., Lee, J.H., Saye-Francisco, K.L., Hsueh, J., Ramos, A., Le, K.M., Jones, M., Jardine, J.G., et al. (2016). A prominent site of antibody vulnerability on HIV envelope incorporates a motif associated with CCR5 binding and its camouflaging glycans. Immunity *45*, 31–45.

Trama, A.M., Moody, M.A., Alam, S.M., Jaeger, F.H., Lockwood, B., Parks, R., Lloyd, K.E., Stolarchuk, C., Scearce, R., Foulger, A., et al. (2014). HIV-1 envelope gp41 antibodies can originate from terminal ileum B cells that share cross-reactivity with commensal bacteria. Cell Host Microbe *16*, 215–226.

Trkola, A., Purtscher, M., Muster, T., Ballaun, C., Buchacher, A., Sullivan, N., Srinivasan, K., Sodroski, J., Moore, J.P., and Katinger, H. (1996). Human monoclonal antibody 2G12 defines a distinctive neutralization epitope on the gp120 glycoprotein of human immunodeficiency virus type 1. J. Virol. *70*, 1100–1108.

Wagh, K., Kreider, E.F., Li, Y., Barbian, H.J., Learn, G.H., Giorgi, E., Hraber, P.T., Decker, T.G., Smith, A.G., Gondim, M.V., et al. (2018). Completeness of HIV-1 envelope glycan shield at transmission determines neutralization breadth. Cell Rep. *25*, 893–908.e7.

Walker, L.M., Huber, M., Doores, K.J., Falkowska, E., Pejchal, R., Julien, J.P., Wang, S.K., Ramos, A., Chan-Hui, P.Y., Moyle, M., et al. (2011). Broad neutralization coverage of HIV by multiple highly potent antibodies. Nature *477*, 466–470.

Wang, S.-K., Liang, P.-H., Astronomo, R.D., Hsu, T.-L., Hsieh, S.-L., Burton, D.R., and Wong, C.-H. (2008). Targeting the carbohydrates on HIV-1: interaction of oligomannose dendrons with human monoclonal antibody 2G12 and DC-SIGN. Proc. Natl. Acad. Sci. *105*, 3690–3695.

Williams, W.B., Liao, H.X., Moody, M.A., Kepler, T.B., Alam, S.M., Gao, F., Wiehe, K., Trama, A.M., Jones, K., Zhang, R., et al. (2015). Diversion of HIV-1 vaccine-induced immunity by gp41-microbiota cross-reactive antibodies. Science *349*, 1–25.

Williams, W.B., Meyerhoff, R.R., Edwards, R.J., Li, H., Manne, K., Nicely, N.I., Henderson, R., Zhou, Y., Janowska, K., Mansouri, K., et al. (2021). Fab-dimerized glycan-reactive antibodies are a structural category of natural antibodies. Cell *184*, 2955–2972.e25.

Yang, Y.Y.M., van Diepen, A., Brzezicka, K., Reichardt, N.-C., and Hokke, C.H. (2018). Glycan microarray-assisted identification of IgG subclass targets in schistosomiasis. Front. Immunol. *9*, 2331.

Zhou, T., Georgiev, I., Wu, X., Yang, Z.-Y., Dai, K., Finzi, A., Do Kwon, Y., Scheid, J.F., Shi, W., Xu, L., et al. (2010). Structural basis for broad and potent neutralization of HIV-1 by antibody VRC01. Science *329*, 811–817.

Zhu, X., Borchers, C., Bienstock, R.J., and Tomer, K.B. (2000). Mass spectrometric characterization of the glycosylation pattern of HIV-gp120 expressed in CHO cells. Biochemistry *39*, 11194–11204.

STAR★METHODS

KEY RESOURCES TABLE

REAGENT or RESOURCE	SOURCE	IDENTIFIER
**Antibodies**		
anti-human-IgG-Cy3	Sigma Aldrich	RRID: AB_258804<br>Cat#: C2571-1ML
PGT128, PGT121, PGT151, PGT130, PGT123	(Walker et al., 2011)	N/A
VRC01	(Zhou et al., 2010)	N/A
F425-b4e8	(Cavacini et al., 2003)	N/A
2G12	(Trkola et al., 1996)	N/A
PCDN76 UCA-S, PCDN76 UCA-P, PCDN76 16-A, PCDN76 22-A, PCDN76 22-C, PCDN76 38-A or PCDN76 38-B	(Macleod et al., 2016)	N/A
Goat α-human-Alexa Fluor 488	Jackson ImmunoResearch	RRID:AB_2337833<br>Cat#:109-545-008
Protein G-HRP	Abcam	Cat#: ab7460
Goat-anti-human IgG H+L 800CW	Li-Cor	Cat#: 926-32232
Goat-anti-human-Fc-AP	Jackson	RRID: AB_2337608<br>Cat#:109-055-098
**Biological samples**		
International AIDS vaccine initiative IAVI Protocol C cohort	(Landais et al., 2016)	N/A
**Chemicals, peptides, and recombinant proteins**		
Biotinylated Phaseolus vulgaris Leucoagglutinin (PHA-L)	Vector laboratories	Cat#: B-1115
Biotinylated Concanavalin A (Con A)	Vector laboratories	Cat#: B-1005
DAPI	Sigma Aldrich	Cat#: D9542-1MG
Rhodamine phalloidin	Life technologies	Cat#: R415
Gp120 (JR-CSF)	This manuscript	N/A
**Critical commercial assays**		
SignalFire™ ECL Reagent	Cell Signaling Technology	Cat#: 6883P3
Human Anti-*Schistosoma mansoni* IgG ELISA Kit	Abcam	Cat#: ab108769
**Experimental models: Organisms/strains**		
Cercariae were shed from Biomphalaria glabrata snails infected with *Schistosoma mansoni* (strain NMRI)	NIAID Schistosomiasis Resource Center	N/A
**Software and algorithms**		
Prism	Graphpad	https://www.graphpad.com/scientific-software/prism/
PyMol	The PyMOL Molecular Graphics System, Version 2.0 Schrödinger, LLC	https://www.pymol.org/
GenePix Pro 6.0 software	Molecular Devices	N/A
Fiji	Image J	N/A
**Other**		
Victor™ X3 multilabel reader	Perkin Elmer	N/A
Agilent G2565BA microarray scanner system	Agilent Technologies, Santa Clara, USA	N/A
Nikon A1 inverted confocal microscope	Nikon Centre, KCL	N/A
Li-Cor Odyssey XF Imaging System	Li-Cor	N/A
ProScanArray® Express	Perkin Elmer, Shelton, USA	N/A
ImageQuant Detector	GE Life Science	N/A

RESOURCE AVAILABILITY

Lead contact

Further information and requests for resources and reagents should be directed to and will be fulfilled by the lead contact, Katie J Doores (katie.doores@kcl.ac.uk).

Materials availability

This study did not generate new unique reagents.

Data and code availability

Microscopy data reported in this paper will be shared by the lead contact upon request.

This paper does not report original code.

Any additional information required to reanalyze the data reported in this paper is available from the lead contact upon request.

EXPERIMENTAL MODEL AND SUBJECT DETAILS

Human subjects

The gender and age of participants in the IAVI Protocol C participants can be found in Table S2.

Ethics for protocol C

The International AIDS vaccine initiative IAVI Protocol C program was conducted between 2006 and 2011 at nine research centers in Kenya, Rwanda, South Africa, Uganda, and Zambia. (Amornkul et al., 2013) The aim was to document and describe immunological, virological and clinical HIV-1 disease progression. A total of 615 participants with documented HIV-1 infection within 12 months were recruited from seroincidence cohorts in Africa. Participants were followed in 3-6 months intervals. Development of broadly neutralizing antibodies (bnAbs) in relation to clinical and virological factors was studied in depth by Landais et al. (Landais et al., 2016). Here, 60 donors were selected to study Schistosoma cross-reactivity in the context of bnAb development. Neutralization breadth was measured on a cross-clade panel of HIV-1 pseudoviruses (92TH021 (CRF0-AE), 94UG103 (Clade A), 92BR020 (Clade B), JRCSF (Clade B), IAVIC22 (Clade C, also named MGRM-C026) and 93IN905 (Clade C)). The level of neutralizing activity of an individual sample was determined by a neutralization score defined as a weighted average of log-transformed 50% neutralization end point dilutions across the reference pseudoviruses neutralization screening panel and excluding the negative and positive con- trols, aMLV and NL43 respectively: (Score = Average (log3 (dilution/100)+1)). All titers below the limit of detection were assigned a value of 33 for purposes of calculating a neutralization score.

The donors selected represent the distribution of the cohort from low neutralization to high (weak (neutralization score <0.5), medium (neutralization score <1 and ≥ 0.5) or top (neutralization score ≥ 1) neutralization scores). To assess longitudinal seroreactvity with *S. mansoni* three timepoints (early, mid-study and late) were chosen. The study was ethically approved by the Republic of Rwanda National Ethics Committee, Emory University Institutional Review Board, University of Zambia Research Ethics Committee, Charing Cross Research Ethics Committee, UVRI Science and Ethics Committee, Kenyatta National Hospital Ethics and Research Committee, KEMRI Scientific and Ethics Review Unit, University of Cape Town Research Ethics Committee, University of Kwazulu-Natal Biomedical Research Ethics Committee, Mahidol University Ethics Committee, Sanford-Burnham Medical Research Institutional Review Board, Veterans Affairs San Diego Institutional Review Board, LIAI Human Subjects Committee, and Scripps Institutional Review Board (Amornkul et al., 2013; Landais et al., 2016). Ethical approval to study pathogen seroreactivity of these samples was granted by King's College London BDM Research Ethics Panel (LRS- 18/19-11221).

METHOD DETAILS

bnAbs incubation on a synthetic glycan microarray

Glycan microarrays were prepared as previously described (Brzezicka et al., 2015; Echeverria et al., 2018). A solution of bnAb (PGT121, PGT123, PGT128, PGT130, PGT151 and PGV04) at 10 μg/mL (final concentration) was incubated with secondary anti-human IgG-Cy3 (1:1000 final dilution, Sigma Aldrich, C2571-1ML) in PBS containing 0.5% bovine serum albumin (BSA) and 0.01% Tween-20 at room temperature for 2 hr in the dark. Glycan microarrays were compartmentalized using an 8 well Proplate^{TM} slide module, 200 μL of the bnAbs/secondary antibody mix was applied to each well and incubated at 4°C overnight under gentle shaking. The microarray slides were washed with PBS+0.01% Tween 20, distilled water and dried in a slide spinner. Fluorescence was analyzed on an Agilent G265BA microarray scanner system (Agilent Technologies, Santa Clara, USA) and quantified with ProScanArray® Express (Perkin Elmer, Shelton, USA) and Microsoft Excel. After local background subtraction the average of mean RFU values and standard deviation of four replicates spots was represented as histograms with Microsoft Excel software.

bnAbs incubation on a shotgun parasite glycan microarray

Glycans were released from glycolipids and glycoproteins from homogenized *S. mansoni* life-stages (cercariae, worms and eggs) by ceramide and PNGaseF treatment, respectively (De Boer et al., 2007; van Diepen et al., 2012, 2015). Glycans were labelled with 2-anthranilic acid (2-AA), fractionated with hydrophobic interaction liquid chromatography (HILIC) and printed on an array slide with an Omnigrid 100 microarrayer (Genomic solutions), as described before. The slides were briefly rinsed with PBS and blocked with 2% BSA in PBS with 50 mM ethanolamine for 1 hour. After a brief rinse with PBST0.05 (0.05% Tween20 in PBS) and PBS, they were diluted in PBST0.01 (0.01% Tween20 in PBS) with 1% BSA and added to the slide and incubated for 1 hr at room temperature. The slide was rinsed with PBST0.05 and PBS. α-human-IgG-Cy3 (1:1,000, Sigma Aldrich, C2571-1ML) in PBST0.01 with 1% BSA was used as secondary antibody and incubated at room temperature for 30 minutes. The slide was washed with PBST0.05, PBS and double distilled H_2O and read on a G2565BA scanner (Agilent Technologies) at 532 nm. The analysis was performed using GenePix Pro 6.0 software (Molecular Devices).

NMR spectroscopy

Compound **28** for STD NMR studies was prepared chemo-enzymatically as previously described (Brzezicka et al., 2015). All experiments were performed at 278 K on a Bruker Avance III 800MHz spectrometer equipped with a 5-mm TXI 800MHz H-C/N-D-05 Z BTO probe. A glycan **28** sample was prepared at 1.4 mM and 30 uM PGT121 in PBS 137 mM at pH 7.4 in D_2O and assigned using standard COSY (cosydfesgpph), TOCSY (mlevphpr), and ^1H-^{13}C HSQC (hsqctgpsp) experiments. The residual water signal was used as a reference for chemical shifts. STD NMR experiments were performed using a train of 50 ms Gaussian pulses (0.4 mW, B_1 field strength 78 Hz) applied on the f2 channel at 0 ppm (on-resonance) or 40 ppm (off resonance). A spoil sequence (2 trim pulses of 2.5 and 5 ms followed by a 40 % z-gradient applied for 3 ms at the beginning of the experiment) was used to destroy unwanted x,y-magnetization from previous scan and a spinlock (1.55 W, 40 ms) was used to suppress protein signals (stddiff.3). To obtain the binding epitopes of the ligands, STD NMR experiments were carried out at different saturation times, D_20, (0.5, 0.75, 1.0, 1.5, 2.0, 2.5, 3.0, 3.5, 4.0, 5.0, and 6.0 s) and the resulting building curves were fitted mathematically to a mono-exponential equation, from which the initial slopes were obtained. From these values, the binding epitope mapping was obtained by dividing all by the largest value, to which an arbitrary value of 100% was assigned.

Molecular docking and molecular dynamics simulations

A molecular model of glycan **28** was produced using the Schrodinger Maestro software, and the conformational flexibility of the model was considered by performing a conformational search using Schrodinger's MacroModel software (Schrödinger, 2016). Conformers were generated by Monte Carlo torsional sampling, eliminating structures with RMSD <5 Å from existing structures or with a potential energy >5 kcal mol^{-1} from the minimum energy structure according to the OPLS3 forcefield. Restraints of 100 kcal mol^{-1} were applied to all internal ring torsions so that the favoured $^4C_1$ ring conformation was preserved along the docking calculation. Generated structures were minimised by the conjugate gradient method, converging on a threshold of 0.05 kcal mol^{-1}. Docking calculations were performed using Schrodinger's Glide software (Friesner et al., 2004; Halgren et al., 2004). A cubic receptor grid was generated around the model of PGT121. Glide docking of glycan **28** conformers was performed using standard precision, enhanced sampling, and a distance-dependent dielectric constant of 2. The resulting poses were clustered using the hierarchical agglomerative average linkage algorithm.

For the molecular dynamics simulations, the complex was parametrised using AMBER ff14SB (Maier et al., 2015) and GLYCAM_06j for protein and carbohydrate moieties respectively. Model systems were generated by solvating with explicit TIP3P water molecules within a truncated octahedron bounding box buffered from the complex by 10 Å and neutralizing with Na^+ ions. Conjugate gradient minimisation was run with 20 kcal mol^{-1} $Å^{-2}$ restraints on solute atoms, converging on a threshold of 1 x 10^{-4} kcal mol^{-1} $Å^{-1}$, before repeating with no restraints. The restraints were reapplied to solute atoms before heating at constant volume to 310 K over a period of 500 ps. The system was then equilibrated at constant pressure (1 atm) over a period of 1.3 ns, with restraints being released slowly over the last 800 ps. In all cases periodic boundary conditions and the particle mesh Ewald method were applied. A Langevin thermostat with a collision frequency of 5 ps^{-1} and a Berendsen barostat with a relaxation time of 2 ps were used. The SHAKE algorithm was used to restrain all bonds involving hydrogen, allowing a timestep of 2 fs to be used. A cutoff of 8 Å was used for non-bonded interactions. For production dynamics, simulations were run for 100 ns, saving coordinates every 10 ps. PBD files of representative frames of the MD simulation represented in Figure 3, panel B are found Data S1–S3.

Measurement of SCA, AWA and gp120 binding by Western blot

Before loading the gels (Mini-PROTEAN® TGX™ Precast Gels 4-20%, BioRad), they were placed in a Mini-PROTEAN Tetra Cell (Bio-Rad) and submerged in running buffer (0.1% SDS, 25 mM tris-Base, 200 mM glycine, pH 8.8). Precision Plus Protein™ Dual Colour standard (BioRad) was loaded on all tris-glycine gels to reference protein sizes and the SDS-PAGE was performed using a power-pack (BioRad) at 200 V until the desired progress was reached. Separated proteins were transferred onto a nitrocellulose membrane (Amersham Protran 0.45), using Mini-PROTEAN Tetra Cell submerged in transfer buffer (20% ethanol, 25 mM Tris-Base and 200 mM Glycine). The transfer was performed at 100 V for 1 hour under cooling and the quality of transfer accessed by brief Ponceau staining (Sigma-Aldrich). The membrane was blocked with 3% BSA in PBS for 1 hour. After the blocking buffer was discarded, the primary antibody (VRC01, F425-b4e8, 2G12, PGT128, PGT121, PGT151) was added (50 μg/mL, diluted in 3% BSA in PBST (1x PBS, 0.05%

Tween20)) and incubated for 1 hour at room temperature with rocking. The membrane was washed three times for 5 minutes in PBST and secondary conjugate added (Protein G-HRP, Abcam, ab7460; 1:1,000, diluted in 5% milk in PBST) and incubated for 1 hr at room temperature with rocking. The membrane was washed 3x (PBST) and SignalFire^{TM} ECL Reagent (Cell Signaling Technology) was used to detect chemiluminescence on an ImageQuant Detector (GE Life Science).

For gp120-JRFL western blot, 0.5 μg of gp120 was loaded in each lane. The membrane was blocked with 3% BSA in PBS for 1 hour. Primary antibody binding (VRC01, F425-b4e8, 2G12, PGT128, PGT121, PGT151 or a bnAb cocktail) was added (50 μg/mL) was detected using secondary antibody (Goat-anti-human IgG H+L 800CW (926-32232, Li-Cor), 1:1,000, diluted in 5% milk in PBST) membrane read using a Li-Cor Odyssey XF Imaging System.

ELISA for SEA

For soluble egg antigen (SEA) ELISAs, the Human Anti-*Schistosoma mansoni* IgG ELISA Kit (ab108769, Abcam) was used and experiments were performed according to the manufacturer's instructions. Human Anti-*Schistosoma mansoni* IgG provided with the kit was used as a positive control. Serum/plasma samples were inactivated with TritonX (0.1%) and diluted to 1:100 before adding to the wells. mAbs were used at 50 μg/mL in sample diluent.

ELISA for SCA and AWA

Soluble cercarial antigen (SCA) and adult worm antigen (AWA) were coated on 96-well Half Area Clear Flat Bottom Polystyrene High Bind Microplates (Corning) at 1 μg per well overnight at 4°C. Wells were washed with PBST and blocked with 5% milk (5% non-fat milk in PBST). Serum/plasma samples were inactivated with TritonX (0.1%) and diluted to 1:100 before adding to the wells. mAbs were used at 50 μg/mL in sample diluent (ab108769, Abcam) and serum standards provided in the Human Anti-*Schistosoma mansoni* IgG ELISA Kit (ab108769, Abcam) were used as controls. The assay was performed with 100 μL per well for all steps, except washing which was performed at 300 μL per well. After the samples were added to the wells the assay plate was incubated at 37°C for 1 hour. After the plate was washed 3x with 1x washing solution, Protein A-HRP solution (provided with the kit) was added. The plate was incubated for 30 minutes at room temperature and washed 3x with washing solution. TMB substrate solution was added to the wells and incubated at room temperature for exactly 15 minutes. The reaction was stopped with stop solution and the OD determined at 450 nm. The kits include a cut-off, positive and negative control. Standard units were determined for serum-samples and are the 10-fold ratio of OD to cut-off OD.

S. mansoni cercariae staining

Live *S. mansoni* cercariae were fixed with 4% para-formaldehyde (PFA) for 20 minutes at room temperature. The cercariae were washed with PBSTx (PBS, 0.3% TritonX100) by allowing them to settle on ice and re-suspension in PBSTx. After permeabilization with 1% SDS in PBS (20 min, room temperature) cercariae were washed with PBSTx and re-suspended in 200 μL staining dilution. The staining dilution was made up with primary HIV-1 antibody at 50 μg/mL (VRC01, F425-b4e8, 2G12, PGT128, PGT121, PGT151, PCDN76 UCA-S, PCDN76 UCA-P, PCDN76 16A, PCDN76 22A, PCDN76 22C, PCDN76 38A, PCDN76 38B) or lectin at 50 μg/mL (PHL-A or Con-A), secondary (Goat α-human-Alexa Fluor 488, Jackson ImmunoResearch; 37.5 μg/mL), DAPI (Sigma, D9542-1MG; 0.1 μg/mL) and Rhodamine phalloidin (Life technologies, R415, used at 1:500). After incubation for 1 hour at 37°C, cercariae were rinsed with PBSTx (3x) and PBS. Cercariae were re-suspended in 7 μL liquefied DAKO mounting medium (Agilent), put on a glass-slide, covered with a coverslip and sealed with clear nail polish. The slides were imaged on a Nikon A1 inverted confocal microscope (Nikon Centre, KCL) and images were analysed with Fiji (Image J) (Collins et al., 2011).

Gp120 ELISA

96-well Half Area Clear Flat Bottom Polystyrene High Bind Microplates (Corning) were coated with gp120-JR-CSF (125 ng per well) in PBS at 4°C overnight. Plates were washed with PBST (1 x PBS, 0.05% Tween20) and blocked with 5% milk (5% non-fat milk in PBST) for 1 hour (RT). The 5% milk was emptied from the wells and antibody dilutions (in 5% milk) added to the wells. Plates were incubated for 2 h at RT and then washed with PBST (5x) and the secondary antibody (anti-human-Fcγ, AP-conjugated; Jackson ImmunoResearch), diluted in 5% milk (1:1,000) was added (1 h, RT, rocking). The plate was washed 5x with PBST and phosphatase substrate (one p-nitrophenyl phosphate tablet dissolved per 5 mL of AP-buffer (10 mM $MgCl_2$, 80 mM $NaCO_3$, 15 mM NaN_3, sterile filtered)) was added to the wells. OD was detected on a spectrophotometer at 405 nm. Curves were plotted using MS Office Excel and GraphPad Prism 8.

QUANTIFICATION AND STATISTICAL ANALYSIS

All ELISA experiments were performed in duplicate. Statistical analyses (Chi-squared test and Mann-Whitney U tests) were performed using GraphPad Prism 8. P-value significances were assigned as described.

Cell Reports, Volume *38*

Supplemental information

Cross-reactivity of glycan-reactive HIV-1 broadly neutralizing antibodies with parasite glycans

Isabella Huettner, Stefanie A. Krumm, Sonia Serna, Katarzyna Brzezicka, Serena Monaco, Samuel Walpole, Angela van Diepen, Fiona Allan, Thomas Hicks, Simon Kimuda, Aidan M. Emery, The IAVI Protocol C Investigators & The IAVI African HIV Research Network, Elise Landais, Cornelis H. Hokke, Jesus Angulo, Niels Reichardt, and Katie J. Doores

Supplementary information

Cross-reactivity of glycan-reactive HIV-1 broadly neutralizing antibodies with parasite glycans.

Isabella Huettner,[1] Stefanie A. Krumm,[1] Sonia Serna,[2] Katarzyna Brzezicka,[2] Serena Monaco,[3] Samuel Walpole,[3] Angela van Diepen,[4] Fiona Allan,[5] Thomas Hicks,[3] Simon Kimuda,[1] Aiden M. Emery,[5] The IAVI Protocol C Investigators & The IAVI African HIV Research Network**, Elise Landais,[6,7] Cornelis H. Hokke,[4] Jesus Angulo,[8] Niels Reichardt,[2,9] Katie J. Doores[1,10]*

[1] Department of Infectious Diseases, School of Immunology & Microbial Sciences, King's College London, London, UK.

[2] Glycotechnology Laboratory, Center for Cooperative Research in Biomaterials (CIC biomaGUNE), Basque Research and Technology Alliance (BRTA), Paseo Miramón 182, 20014 San Sebastian, Spain.

[3] School of Pharmacy, University of East Anglia, Norwich Research Park, Norwich, Norfolk NR4 7TJ, UK.

[4] Department of Parasitology, Leiden University Medical Center, Leiden, the Netherlands.

[5] Department of Life Sciences, Natural History Museum, Cromwell Road, London, United Kingdom.

[6]International AIDS Vaccine Initiative Neutralizing Antibody Center, La Jolla, CA 92037, USA.

[7]International AIDS Vaccine Initiative, New York, NY 10004, USA.

[8]Departamento de Química Orgánica, Universidad de Sevilla, C/ Prof. García González, 1, 41012 Sevilla, Spain; Instituto de Investigaciones Químicas (CSIC-US), Avda. Américo Vespucio, 49, 41092 Sevilla, Spain.

[9]CIBER-BBN, Paseo Miramón 182, 20009 San Sebastian, Spain

[10]Lead contact

* Corresponding author: katie.doores@kcl.ac.uk

**List of investigators and details of affiliations are listed in the acknowledgements

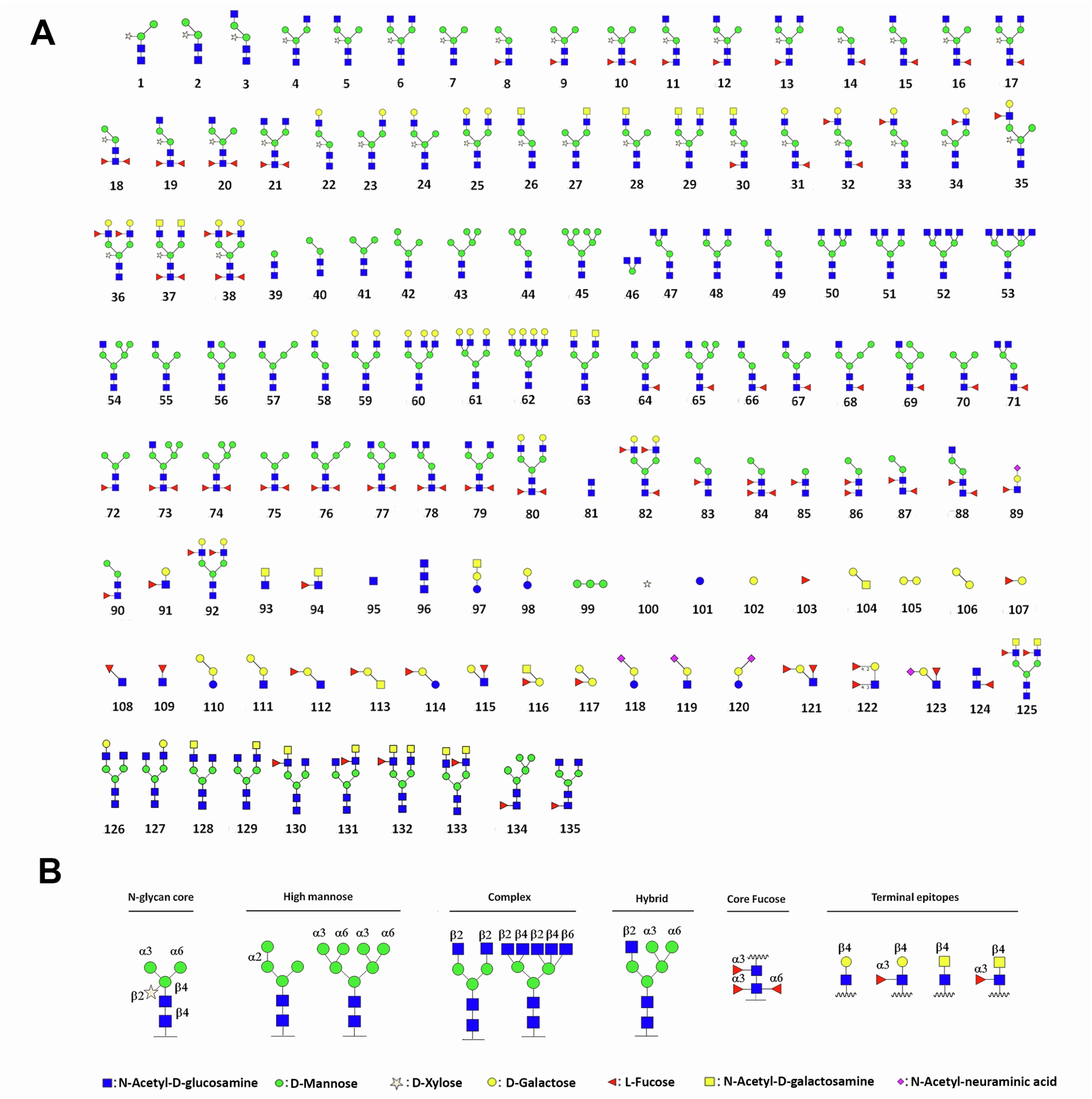

Figure S1: Glycan structures present on the synthetic glycan microarray. A) List of glycan structures present on the synthetic glycan array used in **Figure 1A** and **Figure 2E**. The array used in **Figure 1A** had glycans 1-125 and the array used in **Figure 2E** had been developed further and includes glycans 1-135. **B)** Key for glycan structures shown in panel A. Man: green circle, GlcNAc: blue square, GalNAc: yellow square, Gal: yellow circle, Sialic acid: purple diamond, Fucose: red triangle, and xylose: star. This figure relates to **Figure 1** and **Figure 2E**.

A

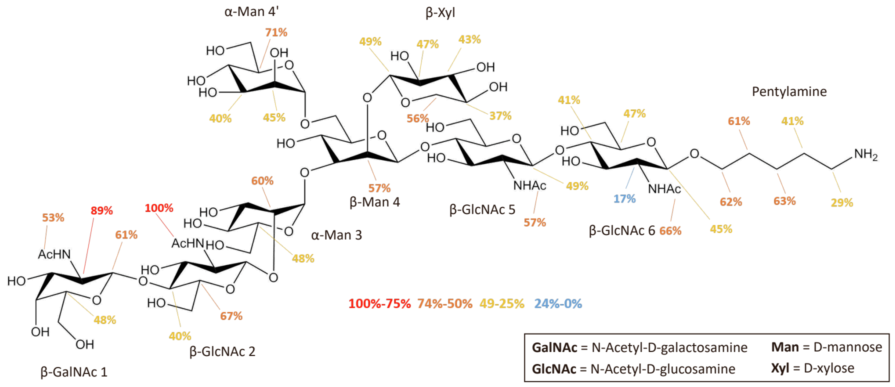
α-Man 4'
β-Xyl
Pentylamine
β-Man 4
α-Man 3
β-GlcNAc 5
β-GlcNAc 6
β-GlcNAc 2
β-GalNAc 1
100%-75% 74%-50% 49-25% 24%-0%
GalNAc = N-Acetyl-D-galactosamine
GlcNAc = N-Acetyl-D-glucosamine
Man = D-mannose
Xyl = D-xylose

B

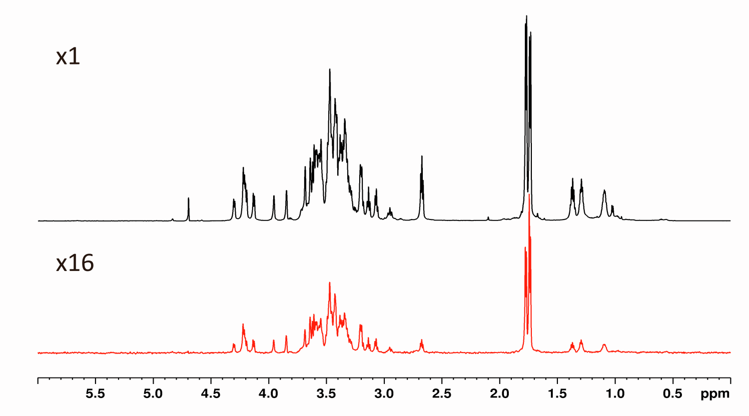
x1
x16
5.5
5.0
4.5
4.0
3.5
3.0
2.5
2.0
1.5
1.0
0.5
ppm

C

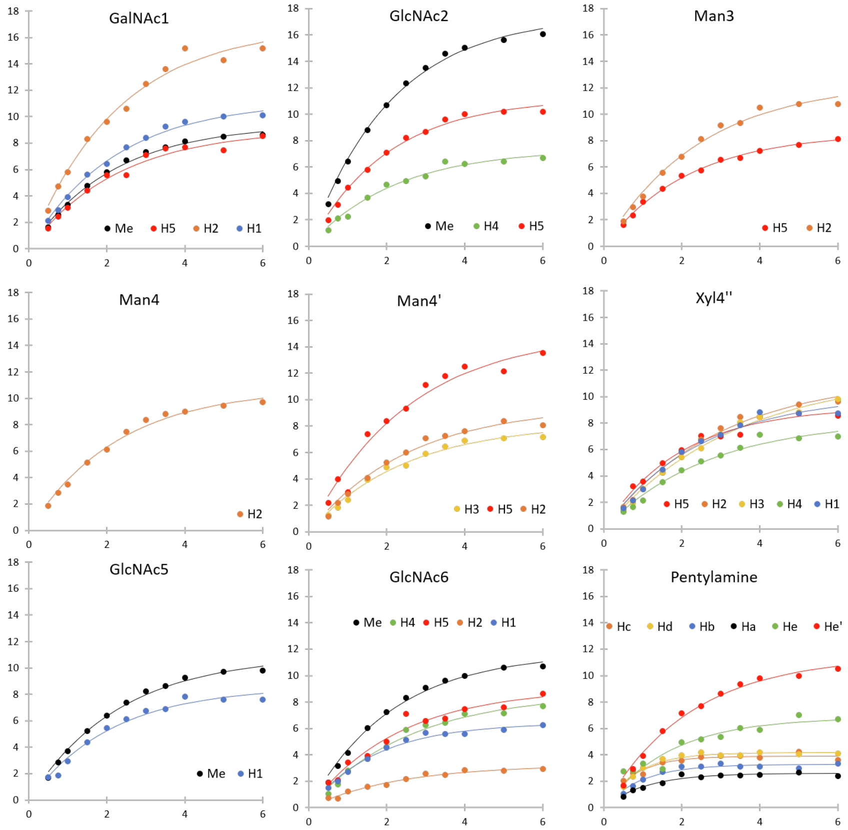
GalNAc1
GlcNAc2
Man3
Man4
Man4'
Xyl4''
GlcNAc5
GlcNAc6
Pentylamine
Me
H1
H2
H3
H4
H5
Hc
Hd
Hb
Ha
He
He'

Figure S2: A) Binding epitope mapping of the parasite glycan 28 as bound to PGT121. Binding epitope mapping of glycan **28** as bound to PGT121 was obtained by mathematically fitting the experimental STD NMR build up curves (see below) to a mono-exponential equation and following an initial slopes analysis. The binding epitope was obtained by normalising all by the largest value, to which an arbitrary value of 100 % was assigned. Hence, higher relative STD % values correspond to closer glycan contacts with PGT121 in the bound state. **B) STD NMR spectra of the glycan 28 / PGT121 complex in solution.** Reference (black) and difference (red) spectra of glycan **28** in the presence of PGT121 at 2 s saturation time. **C) STD NMR build-up curves of the complex of PGT121 with glycan 28**, where the saturation time is represented on the x-axis and the STD% is represented on the y-axis. Build-up curves are obtained for each non-exchangeable hydrogen atom, and they are here grouped by sugar residues. This figure relates to **Figure 2**.

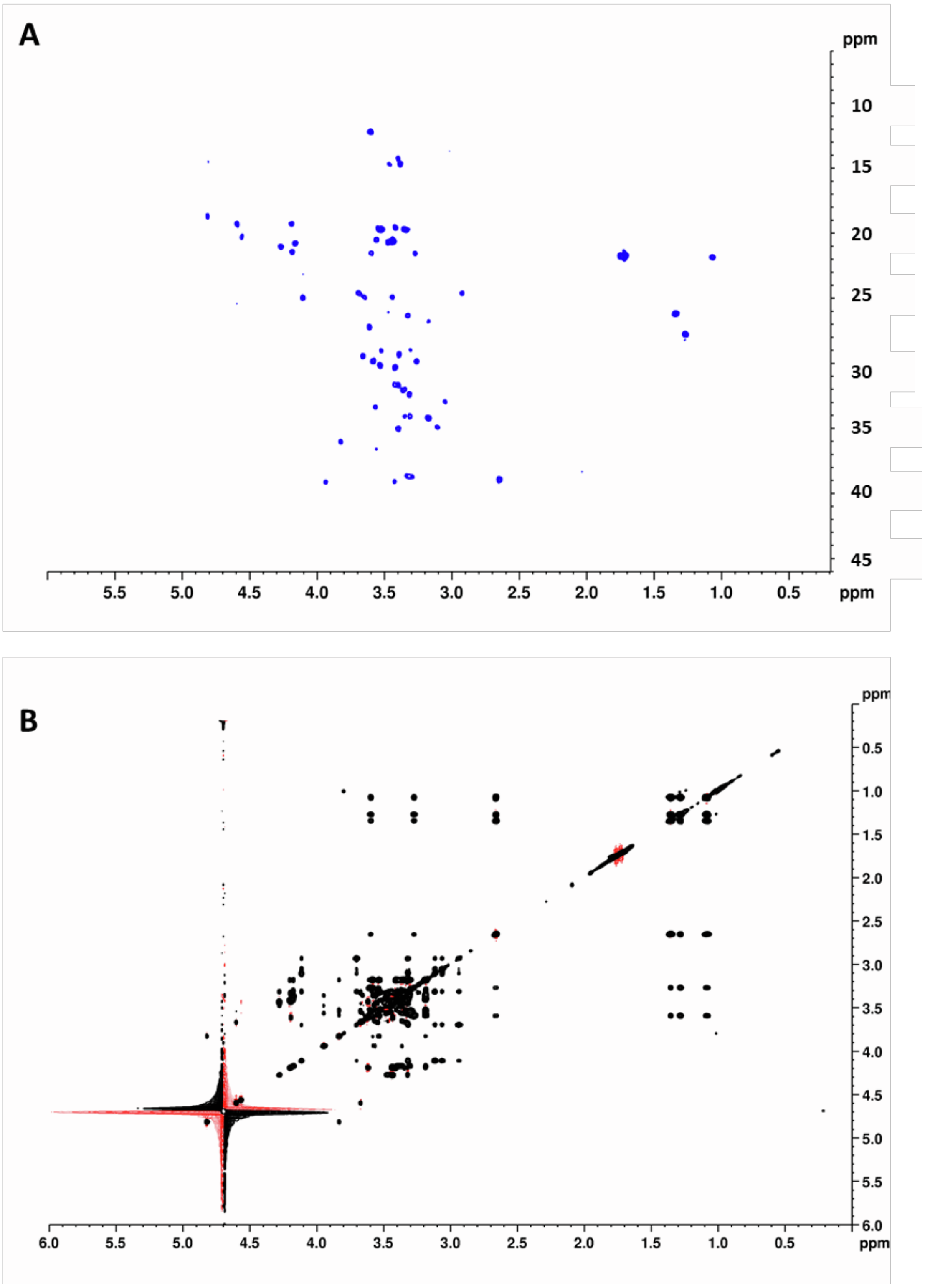

Figure S3: A) HSQC (*hsqcetgpsp*) and B) TOCSY (*mlvevphpp*) of glycan 28 in D_2O, based on which the assignment was based. See Table 2 for full list of assignment. This figure relates to **Figure 2**.

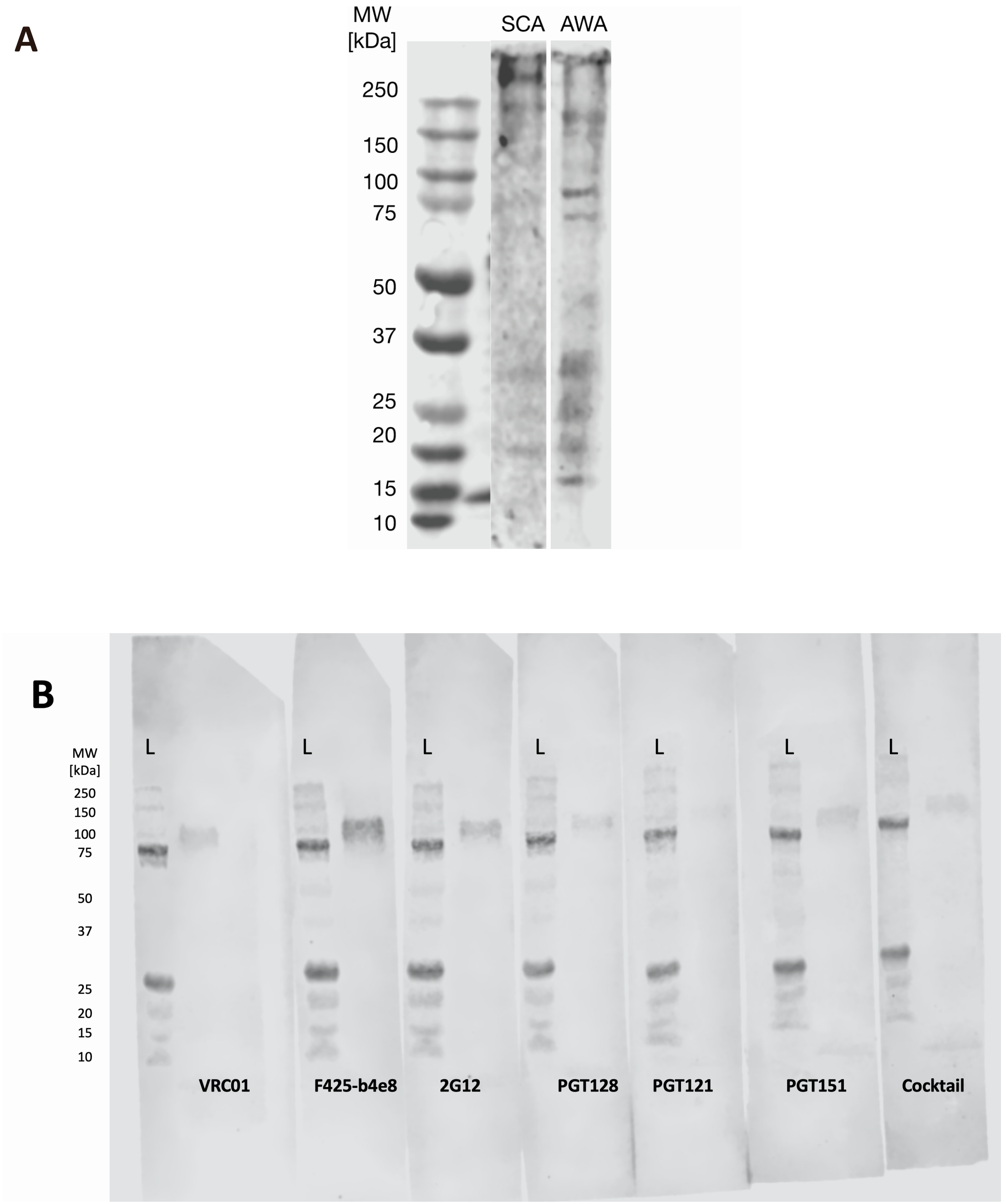

Figure S4: Western blot of SCA/AWA antigens with sera from *S. mansoni* infected individuals and gp120 with HIV-1 bnAbs. A) Binding of sera from *S. mansoni* infected individuals to SCA and AWA by western blot. **B)** Binding to HIV-1 bnAbs (VRC01, F425-b4e8, 2G12, PGT128, PGT121, PGT151 or a cocktail of all mAbs) to recombinant gp120 (JR-FL) by western blot. A denatured, non-reducing gel is used. PGT121, PGT128, PGT151 and 2G12 are all glycan reactive bnAbs. L = ladder. This figure relates to **Figure 3A.**

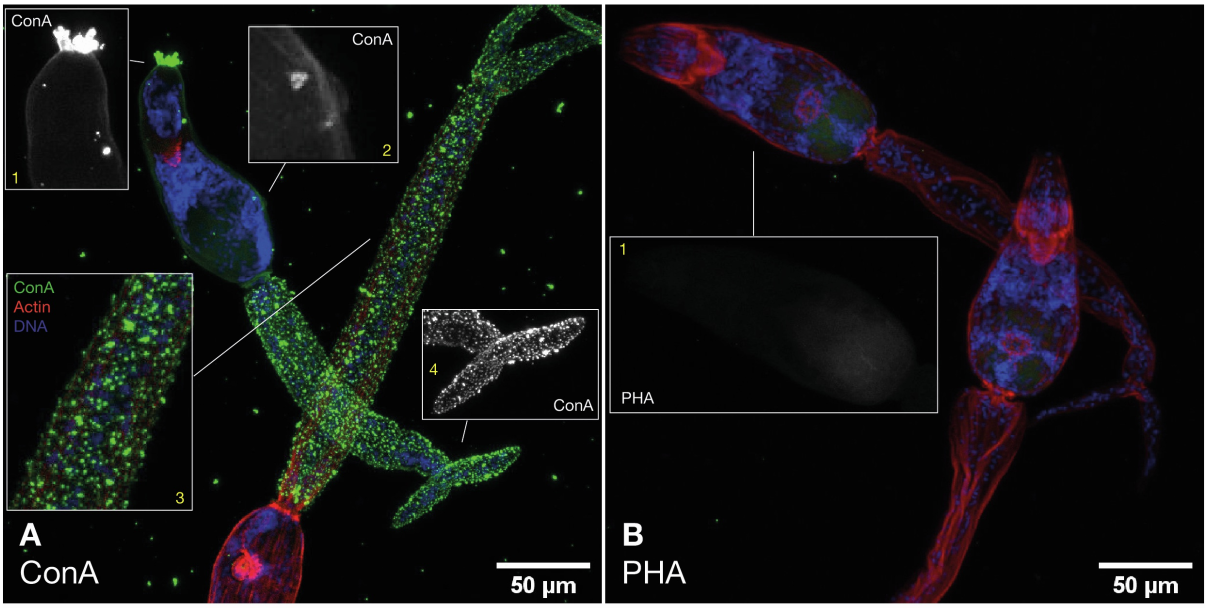

Figure S5: Confocal microscopy images of *S. mansoni* cercariae stained with lectins ConA and PHA-L. Lectins were detected with AlexaFluor488 (green), actin was detected with rhodamine (red) and DNA was stained with DAPI (blue). Black and white images show projections of lectin staining only, while coloured images show overlays. **A)** Confocal microscopy of *S. mansoni* cercariae with ConA, a mannose and glucose binding lectin, which recognizes high-mannose glycans, and some hybrid *N*-glycans. ConA binds to the duct opening on the oral sucker (**1**) and the ventral sucker (**2**). ConA also recognizes surface motifs (spines, ciliated sensory papillae and basement membrane/tegument/glycocalyx structures) above the actin-layer (**3**), and ciliated sensory papillae and basement membrane/tegument/glycocalyx structures (**4**). **B**) Confocal microscopy of *S. mansoni* cercariae with Phytohaemagglutinin-L (PHA-L), a lectin recognizing galactose within tri- and tetra-antennary, complex-type *N*-glycan structures. PHA-L only weakly binds the pre- and post-scetabular gland system (**1**), which is sits interior of the parasite. Confocal microscopy staining was performed twice on different cercariae preparations. Representative images are shown from one experiment. This figure relates to **Figure 3C-F**.

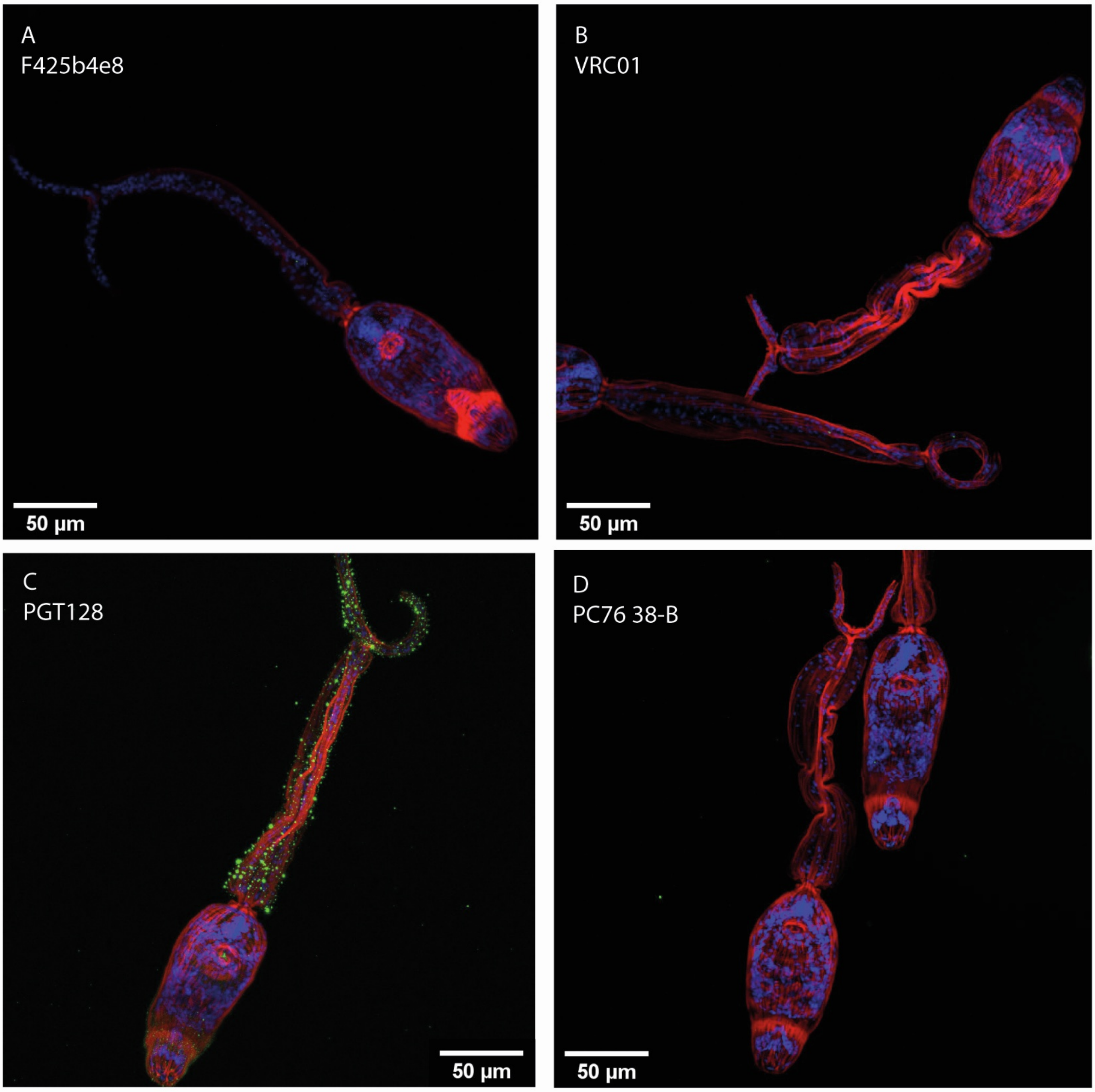

Figure S6: Confocal microscopy images of *S. mansoni* cercariae with control bnAbs and bnAbs not displaying cross-reactivity. Glycan independent HIV-1 bnAbs F425-b4e8 (**A**) and VRC01 (**B**) were used as negative controls. HIV-1 bnAb PGT128 cross-reactivity *S. mansoni* cercariae was determined previously and was included when staining PCDN76 bnAb lineage as a positive control (**C**). PCDN76 clonal variant, 38-B, did not display any cross-reactivity with *S. mansoni* cercariae (**D**). HIV-1 bnAbs were detected with AlexaFluor488 (green), actin was detected with rhodamine (red) and DNA was stained with DAPI (blue). Confocal microscopy staining was performed twice on different cercariae preparations. Representative images are shown from one experiment. This figure relates to **Figures 3C-F** and **Figures 5C-H**.

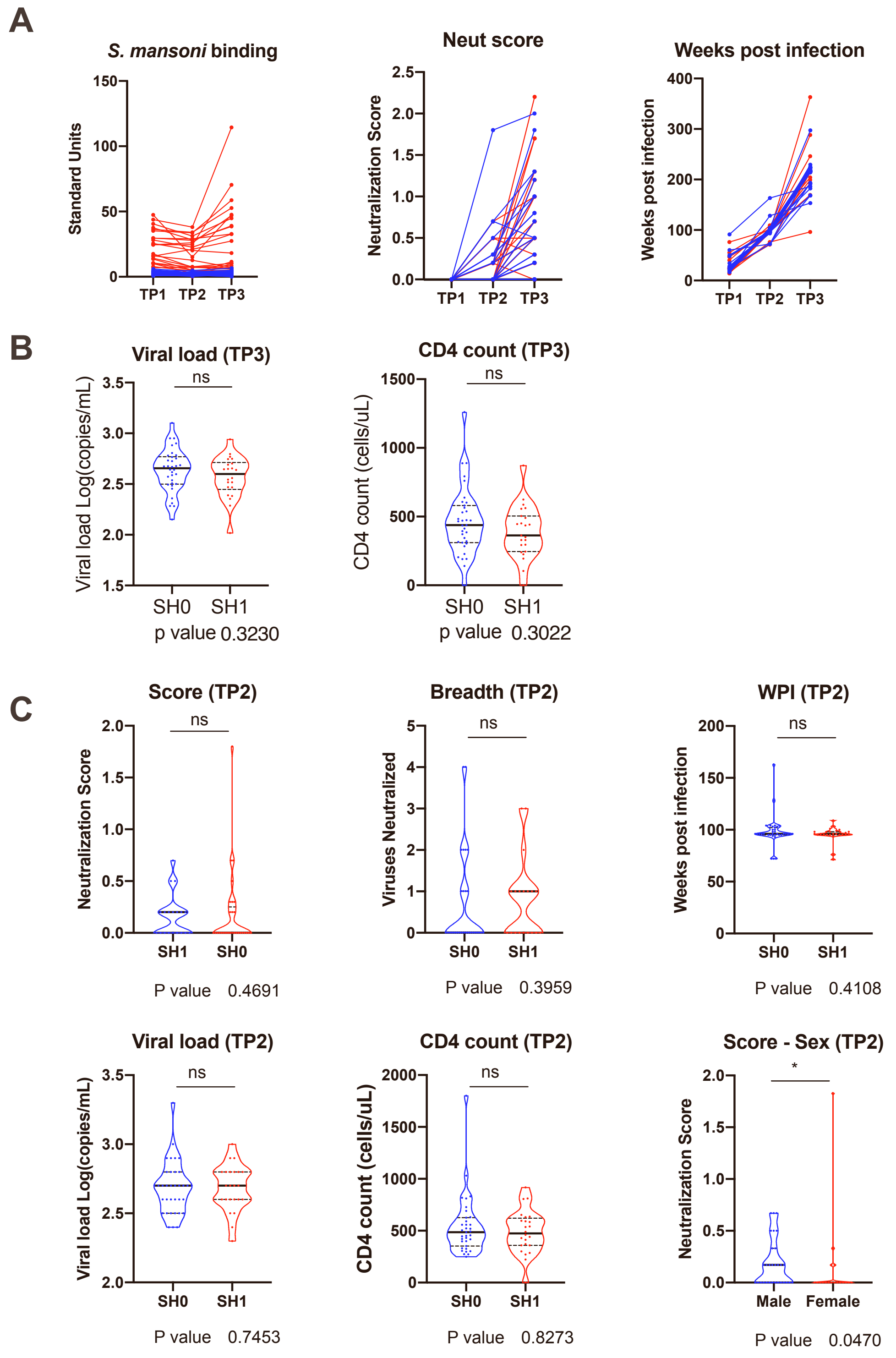

Figure S7: Analysis of HIV-1 neutralization and *S. mansoni* seroprevalence in IAVI Protocol C donors. **A)** *S. mansoni* binding, neutralization score and weeks post infection (WPI) at TP1, TP2 and TP3. SH1 donors are shown in red and SH0 donors are shown in blue. Donors were considered *S. mansoni* seropositive if the SEA binding was above 6.8 Units. **B)** Comparison of CD4 count and viral load at TP3 between donors with *S.*

mansoni seroreactivity (SH1, red) and no *S. mansoni* seroreactivity (SH0, blue). P-values were calculated using Mann-Whitney U tests and significances were assigned as follows: not significant (ns), <0.0332 (*), <0.0021 (**), <0.0002 (***) and <0.00001 (****). **C)** Comparison of neutralization score, the number of viruses neutralized (breadth), weeks post infection, viral load, CD4 count and sex between donors with *S. mansoni* seroreactivity (SH1) and no *S. mansoni* seroreactivity (SH0) at TP2. Statistical differences were determined using Mann-Whitney tests. P-values were reported as follows: not significant (ns), <0.0332 (*), <0.0021 (**), <0.0002 (***) and <0.00001 (****). This figure relates to **Figure 4**.

^{13}C δ	^{1}H δ	Ring identity	Proton identity
**27.756**	1.078	Pentylamine	Hc/Hc'
**27.756**	1.274	Pentylamine	Hd/Hd'
**26.169**	1.346	Pentylamine	Hb/Hb'
**21.763**	1.711	β-GlcNAc6	CH3
**21.763**	1.723	β-GlcNAc2	CH3
**21.763**	1.748	β-GalNAc1	CH3
**21.763**	1.759	β-GlcNAc5	CH3
**38.955**	2.653	Pentylamine	Ha/Ha'
**64.599**	2.93	β-Xyl	H5/H5'
**72.953**	3.054	β-Xyl	H2
**74.899**	3.11	β-Xyl	H3
**66.765**	3.177	Man 3	H4
**74.209**	3.177	β-GlcNAc2	H3
**74.209**	3.18	β-GlcNAc6	H3
**69.83**	3.263	Chain	He/He'
**61.535**	3.28	β-GlcNAc6	H6/H6'
**78.701**	3.301	β-GlcNAc6	H4
**68.951**	3.31	β-Xyl	H4
**74.059**	3.314	β-GlcNAc5	H5
**72.389**	3.322	α-Man 4'	H3?
**66.311**	3.331	β-GlcNAc 5(/b-GalNAc 1)	H6
**78.664**	3.333	β-GlcNAc2	H4
**59.71**	3.349	β-GlcNAc2	H6
**71.983**	3.352	β-Man 4	H4/H5?
**74.059**	3.354	β-GalNAc1	H5
**54.676**	3.391	β-GlcNAc6	H2
**69.303**	3.395	α-Man 4'	H5
**54.237**	3.401	β-GlcNAc 2	H2
**71.671**	3.402	β-Man 4	H4/H5?
**75.032**	3.402	β-GalNAc 1	H4
**59.525**	3.422	β-GlcNAc 5/b-GalNAc 1	H6
**70.32**	3.426	β-GlcNAc 5/b-GalNAc 1	H3
**71.633**	3.426	β-GlcNAc 5/b-GalNAc 1	H3
**79.071**	3.427	β-GlcNAc 5	H4
**64.895**	3.446	β-Man 4	H6/H6'
**60.619**	3.447	α-Man 3	H6/H6'
**54.66**	3.47	β-GlcNAc 5	H2
**60.677**	3.476	β-Man 4	H6
**68.997**	3.529	α-Man 3	H3
**59.71**	3.533	β-GlcNAc2	H6'
**70.146**	3.537	α-Man 4'	H4?
**59.525**	3.547	β-GlcNAc 5(/b-GalNAc 1)	H6
**76.563**	3.564	β-Man 4	H3

**60.619**	3.564	α-Man 3	H6/H6'
**73.347**	3.573	α-Man 3	H5
**69.83**	3.587	Pentylamine	He/He'
**61.535**	3.602	β-GlcNAc6	H6/H6'
**52.162**	3.608	β-GalNAc 1	H2
**67.218**	3.614	β-GlcNAc 2	H5
**64.895**	3.65	β-Man 4	H6/H6'
**69.434**	3.663	α-Man 4'	H2
**64.599**	3.695	β-Xyl	H5/H5'
**76.037**	3.826	α-Man 3	H2
**79.121**	3.94	β-Man 4	H2
**104.929**	4.107	β-Xyl	H1
**100.761**	4.167	β-GlcNAc6	H1
**101.424**	4.185	β-GalNAc 1	H1
**99.282**	4.192	β-GlcNAc 2	H1
**101.021**	4.273	β-GlcNAc 5	H1
**100.236**	4.561	β-Man 4	H1
**99.272**	4.596	α-Man 4'	H1
**98.695**	4.814	α-Man 3	H1

Supplemental Table 1: Glycan 28 ^{1}H and ^{13}C NMR assignment. This table relates to **Figure 2**.

PC Code	Sex	Age at infection	Overall SH Status	Best Neut Score	Score Category	Overall Specificity Mapping	SH sero-reactivity 1	WPI 1	Virus Breadth 2	Neut Score (TP2)	SH sero-reactivity 2	WPI 2	Virus breadth 3	Neut Score (TP3)	SH sero-reactivity 3	WPI 3	ID50 TP3 (Geomean)
PC092	m	30	0	2.2	TOP	Mixed	3.8	24	1	0.17	2.8	96	5	1.83	3.9	218	204
PC064	f	29	0	2.0	TOP	Quaternary Apex	1.4	19	4	1.83	0.3	128	5	2.00	1.4	153	245
PC094	f	29	0	1.7	TOP	Glycan Supersite	3.2	48			1.6	96	4	1.33	2.1	216	97
PC022	f	29	0	1.5	TOP	Glycan Supersite	2.6	91	2	0.33	1.7	163	4	1.33	2.2	186	97
PC035	m	49	0	1.5	TOP	Quaternary Apex	5.3	23	1	0.17	3.0	95	3	1.00	2.3	216	55
PC036	m	24	0	1.3	TOP	Glycan Supersite	0.9	24	2	0.67	1.0	98	5	1.33	0.8	214	118
PC041	f	41	0	1.2	TOP	Interface	4.4	20	0	0.00	2.5	104	4	1.17	2.7	297	80
PC048	f	38	0	1.2	TOP	Interface	3.9	17			0.3	100	5	1.17	2.3	223	98
PC258	f	23	0	1.2	TOP	Mixed	2.4	25			1.3	72	4	1.17	1.3	168	80
PC037	m	20	0	1.2	TOP	Glycan Supersite	3.9	23	1	0.33	4.8	104	3	0.83	6.5	182	102
PC181	m	41	0	1.2	TOP	Glycan Supersite	2.0	24	2	0.50	1.3	96	3	1.00	1.1	168	55
PC082	m	27	0	1.2	TOP	Glycan Supersite	1.5	24	4	0.67	1.6	96	3	0.50	2.5	192	41
PC067	m	23	0	1.0	TOP	Glycan Supersite	5.2	19	1	0.17	2.1	103	2	0.50	1.5	225	26
PC003	m	22	0	0.8	MED	Glycan Supersite	3.4	25			1.8	72	2	0.33	0.5	222	48
PC050	m	24	0	0.8	MED	Interface	2.0	50	2	0.33	2.0	98	4	0.83	0.6	216	73
PC178	f	19	0	0.8	MED	Interface	0.7	24			1.3	96	1	0.33	4.3	191	18
PC049	f	22	0	0.7	MED	Quaternary Kif-	1.6	25	0	0.00	2.1	95	3	0.67	1.8	214	65
PC163	m	45	0	0.7	MED		2.5	23	0	0.00	3.7	96	1	0.33	5.0	216	18
PC144	f	25	0	0.7	MED	CD4bs?	2.0	23	0	0.00	1.3	96	4	0.67	0.0	216	46
PC074	f	20	0	0.5	MED		2.0	60			3.4	72	3	0.50	3.4	193	54
PC034	m	26	0	0.5	MED		1.9	19	0	0.00	1.5	102	1	0.17	2.9	223	19
PC089	m	27	0	0.5	MED		3.2	16	0	0.00	2.5	104	2	0.50	2.9	224	26
PC088	f	26	0	0.3	WEAK		1.9	23	0	0.00	3.1	95	0	0.00	1.7	218	17
PC102	f	31	0	0.3	WEAK		1.2	24	0	0.00	1.0	96	1	0.17	0.2	216	25
PC047	f	25	0	0.3	WEAK		2.7	25	0	0.00	1.0	95	1	0.33	0.7	216	18
PC090	f	46	0	0.3	WEAK		1.5	24	0	0.00	2.1	96	1	0.17	2.7	216	19
PC093	f	42	0	0.3	WEAK		2.7	19	0	0.00	2.0	103	1	0.17	2.0	223	25
PC057	f	21	0	0.2	WEAK		4.2	22	0	0.00	2.8	96	0	0.00	2.9	219	10
PC286	f	28	0	0.2	WEAK		5.9	27	0	0.00	4.2	96	0	0.00	3.4	216	10
PC009	m	33	0	0.2	WEAK		3.0	28	0	0.00	2.1	100	0	0.00	1.7	216	10
PC027	m	24	0	0.2	WEAK		0.8	34	0	0.00	0.7	93	0	0.00	0.8	229	10
PC327	m	39	0	0.2	WEAK		2.2	18	0	0.00	1.5	104	1	0.17	1.4	223	33
PC141	m	50	0	0.2	WEAK		3.2	24	0	0.00	3.0	95	0	0.00	4.3	215	17
PC147	m	24	0	0.0	WEAK		1.1	24	0	0.00	1.0	96	0	0.00	3.2	216	10
PC059	m	32	0	0.0	WEAK		2.1	24	0	0.00	0.6	94	0	0.00	2.9	215	10
PC068	m	33	1	2.3	TOP	Quaternary Kif+	16.0	21	1	0.17	12.7	95	5	2.17	7.2	214	295
PC039	m	27	1	2.0	TOP	Glycan Supersite	7.8	23			4.4	71	5	1.67	5.0	214	223
PC023	m	34	1	1.8	TOP	Interface	4.0	23	0	0.00	2.3	95	5	1.67	11.6	288	170
PC268	m	33	1	1.7	TOP	Glycan Supersite	2.3	15	1	0.17	0.0	101	4	1.67	10.2	169	238
PC174	f	23	1	1.3	TOP	Quaternary Apex	15.8	40	0	0.00	21.0	109	4	1.33	27.4	204	97
PC002	m	22	1	1.2	TOP	Quaternary Kif+	29.7	14	3	0.67	27.9	98	4	1.00	39.1	169	88
PC030	m	44	1	1.2	TOP	Glycan Supersite	24.5	55			24.5	103	5	1.17	70.4	246	98
PC076	m	28	1	1.2	TOP	Glycan Supersite	29.6	47	1	0.17	29.1	95	4	1.00	32.8	216	88
PC029	f	37	1	1.2	TOP	Glycan Supersite	13.6	47	0	0.00	5.0	96	6	1.17	4.3	192	120
PC063	f	34	1	1.0	TOP	CD4bs	10.4	24	0	0.00	5.0	96	4	1.00	2.8	363	88
PC115	f	21	1	0.8	MED		35.2	24	0	0.00	33.7	97	2	0.67	58.7	217	31
PC016	m	28	1	0.8	MED	Quaternary Kif+	40.6	26			31.2	96	2	0.50	44.8	199	44
PC031	m	28	1	0.8	MED	MPER	25.5	24	1	0.17	20.2	98	3	0.67	18.2	216	38
PC043	f	26	1	0.8	MED	Glycan Supersite	14.2	14	1	0.17	7.7	95	3	0.50	10.3	216	71
PC077	m	52	1	0.7	MED	Quaternary Kif-	6.9	76	0	0.00	7.3	100	0	0.00	8.6	217	10
PC079	f	34	1	0.7	MED		36.3	16			34.0	76	2	0.50	52.7	96	34
PC010	m	46	1	0.7	MED		10.6	25	1	0.17	7.3	98	2	0.50	5.9	215	34
PC097	m	29	1	0.7	MED	Quaternary Apex	10.0	47	3	0.50	2.5	96	2	0.33	2.5	215	28
PC018	m	25	1	0.5	MED		43.8	25			38.1	95	1	0.17	114.4	216	33
PC069	m	28	1	0.5	MED	Quaternary Apex	37.6	24	0	0.00	28.3	99	3	0.50	38.6	216	32
PC236	m	26	1	0.5	MED		17.6	23	2	0.50	7.3	95	3	0.50	6.9	217	41
PC101	m	25	1	0.3	WEAK		28.2	22	0	0.00	22.7	94	1	0.17	32.7	216	15
PC038	m	45	1	0.2	WEAK		24.5	24	0	0.00	26.1	96	0	0.00	46.9	216	13
PC107	f	25	1	0.2	WEAK		47.4	24	1	0.17	15.2	95	0	0.00	47.6	222	10

Supplemental Table 2: IAVI Protocol C cohort donors

The PC code represents the donor identifier and sex is reported with ‘m’ for male and ‘f’ for female. Clinical data, neutralization capacity and bnAb specificity was previously determined and reported by Elise Landais and Pascal Poingard and co-workers (Landais et al, 2016). The best overall neutralization score is reported and categorized by ‘TOP’ for top neutralizers (score ≥1), ‘MED’ for medium neutralizers (score <1 and ≥0.5) and ‘WEAK’ for weak neutralizers (score <0.5). Neutralization breadth is reported as number of viruses neutralized out of a 6-virus panel (Landais et al, 2016). Neutralization score (Score) is a numerical representation of neutralization potency of breadth. Geometric mean of ID_{50} as well as *S. mansoni* seroreactivity in standard units and weeks post infection (WPI) are reported for three time points marked as ‘TP1’,’TP2’ and ‘TP3’. Donor PC076 is highlighted in red. This table relates to **Figure 4**.
